# AI-Enabled Wearables for Motor Function Assessment and Rehabilitation in Parkinson Disease: Scoping Review

**DOI:** 10.2196/85596

**Published:** 2026-02-26

**Authors:** Shengting Li, Siqi Chen, Xiaosong Yu, Huixiang Shang, Tingting Tu, Mingtao Quan

**Affiliations:** 1 Department of Nursing Affiliated Hospital of Zunyi Medical College Zunyi, Guizhou China; 2 School of Nursing Zunyi Medical University Zunyi, Guizhou China

**Keywords:** artificial intelligence, Parkinson disease, rehabilitation, scoping review, wearable devices

## Abstract

**Background:**

Artificial intelligence (AI)–enabled wearable devices are rapidly emerging in rehabilitation and motor function assessment for patients with Parkinson disease (PD). However, evidence remains fragmented, integration into nursing practice is limited, and comprehensive synthesis is lacking.

**Objective:**

This study aimed to summarize studies on AI-enabled wearable devices for PD rehabilitation and motor function assessment, describing device types, monitored indicators, algorithms, and application characteristics, and identifying research gaps and barriers to clinical translation.

**Methods:**

Guided by the PRISMA-ScR (Preferred Reporting Items for Systematic Reviews and Meta-Analyses extension for Scoping Reviews) framework, 9 databases (China National Knowledge Infrastructure, Wanfang Data, SinoMed, Cochrane Library, PubMed, Web of Science, CINAHL, Scopus, and Embase) were searched from inception to December 2025. Eligible studies were published in English or Chinese from January 1, 2020, onward and enrolled people with PD using noninvasive, body-worn AI-enabled wearable devices for rehabilitation, assessment, or monitoring. Dissertations and full conference papers were included, whereas preprints and conference abstracts were excluded. Methodological quality was appraised using the Mixed Methods Appraisal Tool, 2018 tool. Results were synthesized narratively and mapped to characterize devices, sensing modalities, algorithms, and evaluation methods.

**Results:**

A total of 66 studies involving approximately 3579 participants were included. Wearable devices mainly comprised multisensor modules, smart insoles, and wrist-worn devices, with accelerometers being the most frequently used sensors. Data collection was predominantly passive, and most studies were conducted in laboratory or clinical settings using single- or short-term sessions. Internal validation approaches, particularly leave-one-out and k-fold cross-validation, were common, whereas external validation was rare, and reporting of calibration and clinical decision thresholds was limited. Sensitivity and accuracy were the most frequently reported performance metrics, highlighting substantial heterogeneity in analytical methods and outcome reporting.

**Conclusions:**

This scoping review systematically synthesized evidence on AI-enabled wearable devices for motor function assessment and rehabilitation in PD, complemented by an evidence map and guided by a rehabilitation- and nursing-oriented perspective, and identified key translational gaps between proof-of-concept studies and real-world rehabilitation workflows. Compared with previous reviews that primarily focused on monitoring functions or device performance, this review places greater emphasis on rehabilitation applications and nurse-led translation into practice, and proposes a conceptual “challenges and opportunities” framework to inform the design, evaluation, and reporting of devices and algorithms, while further highlighting key considerations for workflow integration and the implementation of decision-support systems. These findings have practical relevance for advancing continuity of rehabilitation across clinical, home, and community settings, and may help guide nurses in delivering continuous monitoring, personalized follow-up, and timely intervention, thereby improving the efficiency and accessibility of rehabilitation management.

## Introduction

### Background

Parkinson disease (PD) is a common neurodegenerative disorder characterized by tremor, bradykinesia, rigidity, and postural or gait disturbances [1,2]. Its high prevalence and disability rates profoundly impair patients’ quality of life and impose substantial burdens on families and health care systems. Since 1990, the number of patients with PD has more than doubled—from approximately 2.8 million to over 6.2 million—and is projected to exceed 12 million by 2040 [3]. In China, accelerated population aging has led to a rising prevalence, with an estimated 1.37% among adults aged 60 years and older and more than 3.62 million patients nationwide [4,5]. Rehabilitation remains essential for improving motor function and slowing functional decline [6-8]. However, conventional face-to-face rehabilitation is time- and resource-intensive, often inaccessible in remote regions, and frequently associated with poor adherence [9,10].

In recent years, the rapid development and application of wearable devices have provided significant advantages for assessment and intervention in neurological and psychiatric disorders, such as stroke and depression [11,12]. Wearable devices encompass a wide range of intelligent electronic devices, including smartwatches, wristbands, insoles, exoskeletons, multisensor wearable devices, and electronic textiles [13-16]. These devices support continuous, real-time, and multidimensional monitoring during daily activities or rehabilitation training, capturing key motor indicators such as gait, tremor, joint mobility, postural control, motor coordination, and overall physical activity levels [17-19].

Against this backdrop, the rapid emergence of artificial intelligence (AI)–enabled wearable devices has created new opportunities for the rehabilitation of PD. In this review, AI-enabled wearable devices refer to noninvasive wearable technologies that incorporate machine learning (ML) or deep learning (DL) methods for data-driven analysis beyond rule-based approaches. Unlike traditional approaches that rely on manual observation or intermittent scale-based assessments, AI-enabled wearable devices enable the capture of large volumes of fine-grained data. Supported by ML and DL techniques, these data can be efficiently processed and intelligently interpreted within a data-driven rehabilitation framework, highlighting the importance of AI model interpretability for clinical and nursing decision-making [20-22]. Moreover, AI-enabled wearable devices facilitate continuous remote monitoring and long-term follow-up, making it possible to track disease progression and changes in motor function in real time within home-based rehabilitation settings. These data can then be leveraged by clinicians and rehabilitation teams to design more precise training programs [23,24]. Such an approach enhances the continuity and personalized rehabilitation, offers a feasible pathway for remote and home-based rehabilitation, and holds promising potential for optimizing health care resource allocation and improving patient adherence.

### Research Questions and Objectives

Although numerous studies and reviews have explored the use of wearable devices in PD rehabilitation, several limitations remain, including (1) most studies involve small sample sizes and short follow-up periods, providing insufficient longitudinal evidence to evaluate long-term rehabilitation outcomes [17,25,26]; (2) current reviews mainly focus on device performance or symptom monitoring, with limited attention to AI data-processing workflows, algorithmic applications, and their potential value for clinical and nursing practice [18,19]; (3) studies differ substantially in device types, monitoring indicators, data-processing strategies, and AI algorithm selection. The absence of a unifying framework to synthesize these variations hinders a comprehensive understanding of the research landscape [27-29]; and (4) evidence in nursing practice remains limited; despite nurses’ central role in rehabilitation, research on AI-enabled tools for nursing decision-making, health education, and adherence support is still scarce [30,31]. Collectively, these issues result in fragmented and unsystematic evidence, limiting its ability to guide clinical rehabilitation nursing practice and theoretical development.

To address these needs, we conducted a scoping review and developed an evidence map to systematically present the current applications of AI-enabled wearable devices in PD rehabilitation and motor function assessment. The scoping review summarized the breadth, depth, and nature of existing evidence [32], while the evidence map visualizes research distribution, patterns, and emerging trends across domains [33]. By integrating these 2 approaches, this study aimed to map and visualize the current evidence on AI-enabled wearable devices for PD rehabilitation and motor function assessment. The objectives were to summarize device types, monitoring indicators, AI algorithms, and application characteristics, and to identify research gaps and future directions to support nursing practice and further digital-health implementation in PD rehabilitation.

## Methods

### Protocol and Registration

The review followed the methodological guidance outlined in the PRISMA-ScR (Preferred Reporting Items for Systematic Reviews and Meta-Analyses extension for Scoping Reviews) [34], and the completed checklist is provided in Multimedia Appendix 1. No protocol was registered for this review.

### Eligibility Criteria

The inclusion criteria were defined using the population, concept, and context framework. Eligible studies enrolled participants with a confirmed diagnosis of PD and used noninvasive, body-worn wearable devices integrated with AI-based analytical methods. The focus of included studies was on rehabilitation, motor function assessment, or monitoring. Gray literature such as dissertations and full conference papers was included, whereas preprints and conference abstracts were excluded to ensure methodological consistency and to prioritize evidence that had undergone formal peer review. Only studies published in English or Chinese on or after January 1, 2020, were eligible for inclusion. Detailed inclusion and exclusion criteria are presented in Multimedia Appendix 2.

### Information Sources

A comprehensive search was conducted on August 12, 2025, across 9 databases: CNKI (China National Knowledge Infrastructure), Wanfang Data, SinoMed, Cochrane Library, PubMed, Web of Science, CINAHL, Scopus, and Embase. The literature coverage spanned from database inception to the search date. No websites or other nondatabase information sources were searched. Reference lists and forward citations of the included studies were additionally screened to identify further potentially relevant records. To ensure the currency and completeness of the evidence, an updated search using the same strategy was conducted on December 10, 2025.

### Search Strategy

The reporting of the search process adhered to the PRISMA-S (Preferred Reporting Items for Systematic Reviews and Meta-Analyses literature search extension) [35]. The search strategy was developed by a researcher trained in systematic literature retrieval and combined MeSH (Medical Subject Headings) terms with free-text keywords across four core concepts: (1) wearable or body-worn mobile devices; (2) rehabilitation, assessment, or monitoring; (3) PD; and (4) AI or ML. No limits or restrictions, including language, publication date, or study design, were applied at the search level. All eligibility criteria were applied manually during title/abstract screening and full-text review. The search strategy did not undergo peer review. Complete search strategies for each database and information source are provided in Multimedia Appendix 3.

### Selection of Sources of Evidence

The selection process comprised two stages: (1) title/abstract screening and (2) full-text review. In the first stage, 1 reviewer imported all records into EndNote X9 (version 12062; Clarivate), removed duplicates, and excluded studies outside the eligible date range. Two reviewers then independently screened titles and abstracts to determine provisional inclusion. In the second stage, the same 2 reviewers independently assessed the full texts to establish the final set of included studies. Disagreements were resolved through discussion; unresolved cases were adjudicated by a third reviewer. Cohen κ was calculated to measure the interrater agreement [36]. Agreement was high for title and abstract screening (κ=0.86), whereas it was moderate for full-text review (κ=0.57).

### Data Charting Process

Data charting was conducted independently by 1 reviewer using a standardized extraction form developed a priori. The form included key study characteristics, wearable device details, AI methods, outcomes, and findings relevant to the review questions. Following Joanna Briggs Institute guidance, charting was iterative, and the form was refined as needed. A second reviewer verified all data, with disagreements resolved by a third reviewer. The form was pilot-tested on 5 studies before full implementation.

### Data Items

The data extraction form was developed by the research team based on the objectives of this review and included three core modules: (1) study details, (2) wearable device details, and (3) AI details. When the relevant information was not reported, it was recorded as not available. Each module contained specific data variables, as detailed in Multimedia Appendix 4.

### Critical Appraisal of Individual Sources of Evidence

The methodological quality of all included studies was evaluated using the Mixed Methods Appraisal Tool, 2018 version [37]. Two reviewers conducted the assessments independently and resolved discrepancies through discussion; cases in which consensus could not be reached were adjudicated by a third reviewer. The appraisal was used descriptively to identify methodological strengths and limitations of the included studies and was not used as a criterion for study inclusion. The detailed appraisal results are provided in Multimedia Appendix 5 [38-103].

### Synthesis of Results

A narrative synthesis approach was used. Extracted data were summarized thematically and presented in descriptive text, tables, and an evidence map to illustrate research trends, methodological characteristics, and application domains of AI-enabled wearable devices in PD.

### Ethical Considerations

This scoping review used only published, peer-reviewed literature and therefore did not require approval from an ethics committee or institutional review board. Findings will be submitted to an open access, peer-reviewed journal and presented at relevant medical and engineering conferences.

## Results

### Study Selection

**Figure 1:**
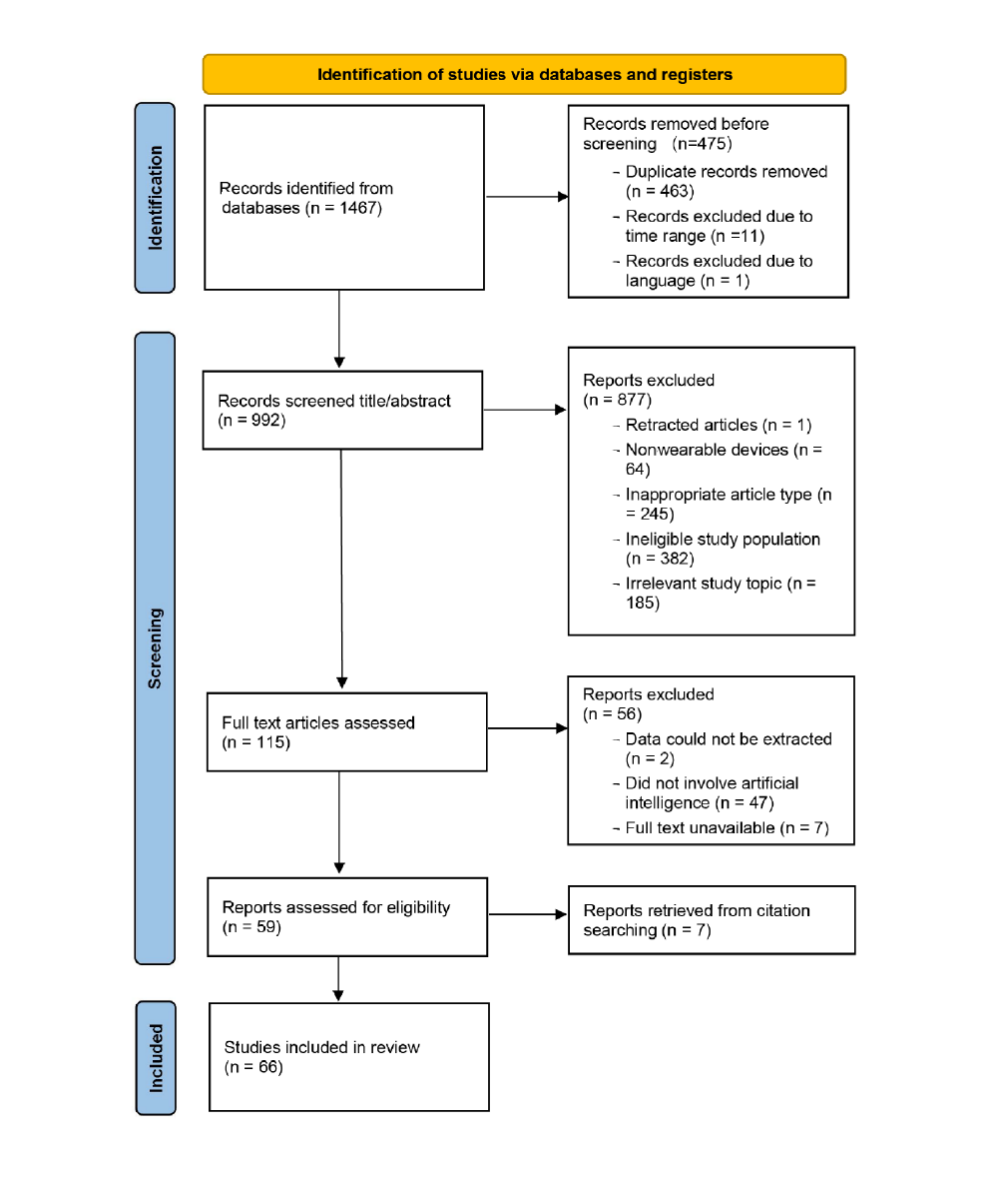
Flowchart of the study selection process.

A total of 1467 records were initially retrieved. Of these, 475 (32.38%) were removed using EndNote X9 due to duplication, publication date, or language incompatibility. The remaining 992 (67.62%) records underwent title and abstract screening, during which 877 (88.41%) were excluded. Among the 115 records reviewed in full text, 47 (40.87%) were unrelated to AI, 7 (6.09%) were unavailable in full text, and 2 (1.74%) lacked extractable data. An additional 7 relevant studies were identified through reference tracing. In total, 66 studies were included in this review [38-103]. The detailed selection process is illustrated in the PRISMA (Preferred Reporting Items for Systematic Reviews and Meta-Analyses) flow diagram (Figure 1).

### Characteristics of Included Studies

**Figure 2:**
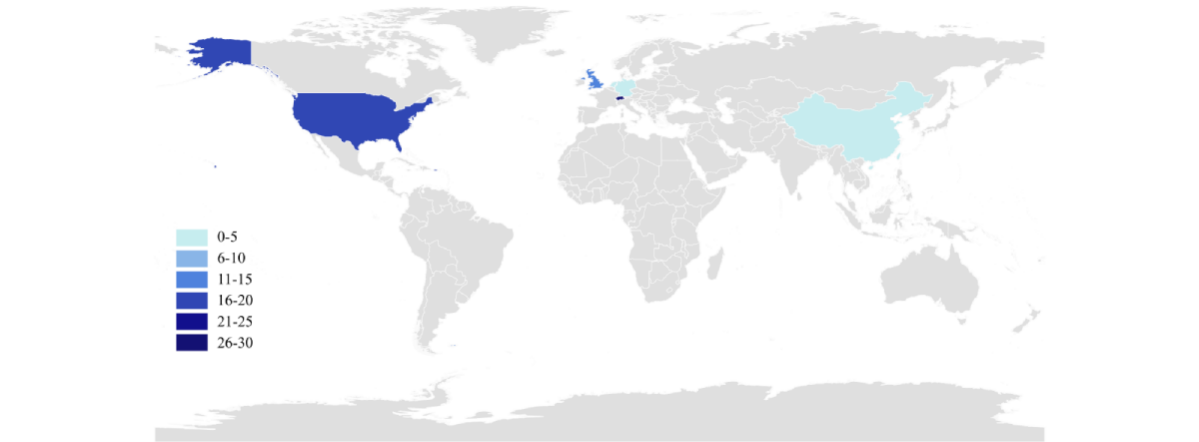
Geographical distribution of the included studies using artificial intelligence–enabled wearable devices for Parkinson disease.

All included studies were published between 2020 and 2025, and the publication volume remained relatively stable over this 6-year period. The highest number of studies was published in 2025 (16/66, 24.24%; Table 1). The included studies originated from multiple countries and regions (Figure 2), with Switzerland contributing the largest proportion (27/66, 40.91%). With respect to publication type, the vast majority were journal articles (61/66, 92.42%).

**Table 1:** Characteristics of the included studies using artificial intelligence–enabled wearable devices for Parkinson disease (PD; N=66).

Features		Values		References
**Year of publication, n (%)**				
	2020	8 (12.12)	[38,47,50,53,61,63,66,73]	
	2021	11 (16.67)	[44,49,55,56,59,62,72,82,86,94,96]	
	2022	9 (13.64)	[39,46,51,58,64,68,80,81,83]	
	2023	11 (16.67)	[43,57,60,71,74,76,77,84,85,89,91]	
	2024	11 (16.67)	[48,54,65,67,69,70,78,87,90,93,95]	
	2025	16 (24.24)	[40-42,45,52,75,79,88,92,97-103]	
**Type of publication, n (%)**				
	Journal article	61 (92.42)	[38,40-45,47-69,71-84,86-91,93-103]	
	Conference paper	4 (6.06)	[39,46,70,92]	
	Thesis	1 (1.52)	[85]	
**Country/region of publication, n (%)**				
	Switzerland	27 (40.91)	[41-44,47,49,55,57,58,62,65-68,71,75,79,80,88,90,91,96,97,99,101-103]	
	United States	19 (28.79)	[39,46,48,51,54,59,61,64,69,70,73,74,76,78,81,86,89,92,94]	
	United Kingdom	11 (16.67)	[50,52,53,56,60,63,72,87,93,95,98]	
	Netherlands	5 (7.58)	[38,40,45,82,100]	
	China	3 (4.55)	[83-85]	
	Germany	1 (1.52)	[77]	
**Number of participants**				
	Mean (SD; range)	55.06 (132.78; 2-1057)	[38-99,101-103]	
	1-30, n (%)	41 (62.12)	[38-42,44,46-49,52,55-64,67,69-75,82,85,86,90,91,93-97,99,103]	
	31-60, n (%)	8 (12.12)	[43,68,76-79,81,92]	
	61-100, n (%)	9 (13.64)	[45,50,51,53,65,66,83,89,101]	
	101-500, n (%)	6 (9.09)	[54,84,87,88,98,102]	
	>500, n (%)	1 (1.52)	[80]	
	Not reported	1 (1.52)	[100]	
**Age distribution of participants^a^ (years)**				
	Mean^b^ (SD; range)	64.59 (10.03; 25.00-76.10)	[38-47,49-53,56-61,63-76,80,81,83-85,89,91-93,95,96,98,99,101-103]	
	<60, n (%)	3 (4.55)	[39,40,55]	
	60-69, n (%)	39 (59.09)	[42-45,47,49,51,53,56,63,65-67,69-77,79-81,83,85,86,88,89,91-93,95,96,98,99,102,103]	
	70-79, n (%)	14 (21.21)	[38,41,46,50,52,57-61,64,68,84,101]	
	≥80, n (%)	2 (3.03)	[54,97]	
	Not reported	8 (12.12)	[48,62,78,82,87,90,94,100]	
**Sex distribution^c^**				
	Female (%), mean (SD; range)	35.88 (18.08; 0–100)	[38-41,45-48,50-68,70-73,75,79-81,83-92,94-96,98,99,101,102]	
**Inclusion details n (%)**				
	PD only	47 (71.21)	[38,40,41,43,46-49,51,52,54-72,76,81,82,84-88,90,93-99,102,103]	
	Mixed	18 (27.27)	[39,42,44,45,50,53,73-75,77-80,83,89,91,92,101]	
	Not reported	1 (1.52)	[100]	
**Application objective^d^, n (%)**				
	Motor function assessment	54 (81.82)	[38,39,41-47,49,50,52-55,57-80,82-89,91,92,95,99-102]	
	Disease progression/symptom monitoring	26 (39.39)	[39,46,51-53,55,56,65,68,69,71,74,76,79,87,89,90,93,94,96-100,102,103]	
	Efficacy evaluation	11 (16.67)	[46,54,56,61,70,71,77,78,81,90,103]	
	Rehabilitation training	8 (12.12)	[40,48,54,55,60,67,77,96]	
	Others	1 (1.52)	[88]	

^a^Reported as mean (SD), median (IQR), or range, depending on the study.

^b^Age was summarized from studies reporting mean (SD); studies with only median or without age were excluded. For mixed populations, only PD patient data were used.

^c^Based only on studies that reported sex data.

^d^Application objectives were classified based on the primary functional aim of the wearable system. Motor function assessment refers to quantitative evaluation of motor performance at specific time points; disease progression/symptom monitoring refers to continuous or longitudinal tracking of disease states; efficacy evaluation refers to the assessment of responses to interventions or treatments; and rehabilitation training refers to systems explicitly designed to support training or feedback to improve motor function. The number of studies does not add up, as several studies have used >1 application objective.

Regarding study populations, sample sizes ranged from 2 to 1057 participants, with a mean sample size of 55.06 (SD 132.78), yielding a total of approximately 3579 participants. Overall, most studies involved relatively small samples with substantial heterogeneity, and the majority included only patients with PD (47/66, 71.21%). In terms of the application objectives of wearable devices, most studies focused on motor function assessment (54/66, 81.82%), whereas rehabilitation-oriented applications were relatively scarce, being reported in only 8 (12.12%) studies. Overall, this distribution suggests that intervention-focused research remains limited. Detailed characteristics of the included studies are presented in Table 1 and Multimedia Appendix 6 [38-103]. For categories in which studies could contribute to more than 1 classification, percentages may exceed 100%; detailed coding rules are provided in the table footnotes.

### Characteristics of Wearable Devices

**Figure 3:**
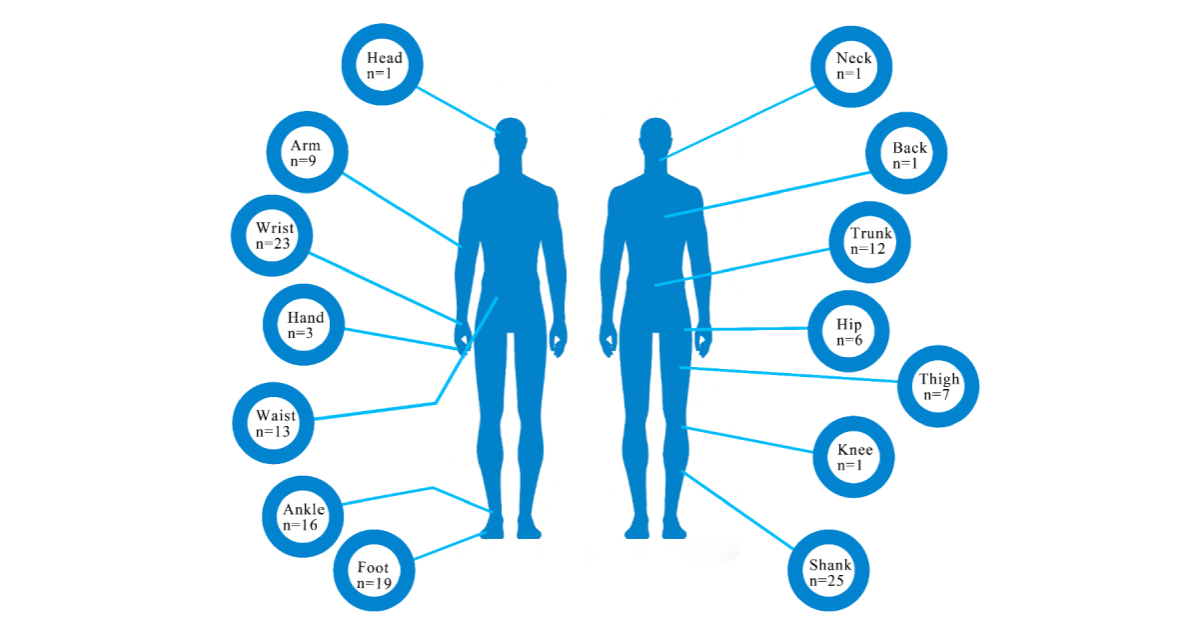
Placement of wearable sensors in studies using artificial intelligence–enabled wearable devices for Parkinson disease.

A total of 5 major types of wearable devices were identified across the included studies. Sensor module was the predominant device type, accounting for 84.85% (56/66) of studies, followed by smart insoles (7/66, 10.61%) and smartwatches (5/66, 7.58%; Table 2). Regarding device origin, commercially available devices were more common (41/66, 62.12%) than noncommercial devices (21/66, 31.82%); nevertheless, a substantial proportion of studies still relied on customized or research-grade devices. A total of 14 distinct wearing locations were reported (Figure 3). The most common placements were the shank (25/66, 37.88%) and the wrist (23/66, 34.85%), followed by the foot (19/66, 28.79%) and the ankle (16/66, 24.24%). Many studies used multiple wearing locations within a single study, reflecting the diversity of measurement strategies adopted.

**Table 2:** Features of artificial intelligence–enabled WDsa used in the included Parkinson disease studies (N=66).

Features		Values, n (%)	References
**Type of WD^a,b^**			
	Sensor module	56 (84.85)	[38-45,47,48,50-75,78-82,84,86-89,91-98,102,103]
	Smart insole	7 (10.61)	[39,46,55,57,59,100,101]
	Smartwatch	5 (7.58)	[49,63,85,97,99]
	Smart wristband	3 (4.55)	[83,85,90]
	Stimulator	2 (3.03)	[48,103]
	Others (adhesive electrodes, ankle band, or smart glove)	3 (4.55)	[42,76,77]
**Status of WD^a,b^**			
	Commercial	41 (62.12)	[38,42,44,46,48,49,51-59,63-67,70-72,74,75,80,81,84-89,91-93,97-99,101,102]
	Noncommercial	21 (31.82)	[39-41,43,45,47,48,50,60,67-69,73,76-78,83,84,90,100,103]
	Not reported	8 (12.12)	[61,62,79,82,85,94-96]
**Company of WD^a,b^**			
	APDM Inc	6 (9.09)	[53,62,64,66,86,87]
	Shimmer Sensing	5 (7.58)	[38,55,59,85,93]
	Activinsights Ltd	4 (6.06)	[43,44,85,97]
	MC10 Inc	3 (4.55)	[53,63,75]
	Moticon ReGo AG	3 (4.55)	[46,57,101]
	Noraxon USA Inc	2 (3.03)	[42,74]
	PD Neurotechnology Ltd	2 (3.03)	[57,71]
	Great Lakes NeuroTechnologies Inc	2 (3.03)	[56,70]
	STMicroelectronics	2 (3.03)	[68,69]
	Tekscan Inc	2 (3.03)	[55,59]
	Self-developed	13 (19.70)	[39,51,60,61,67,73,76,77,83,84,89,100,103]
	Others	16 (24.24)	[43,44,48,50,54,58,63,65,88-92,96,99,102]
	Not reported	16 (24.24)	[40,41,45,47,49,52,72,78-82,94,95,97,98]
**Placement^b^**			
	Shank	25 (37.88)	[40,42-44,48,52,55,59,60,67,69,71,74,75,80-82,84,86,88,91,93-95,102]
	Wrist	23 (34.85)	[38,44,49,53,56-58,63,69-71,79,83,85,87-92,97,99,102]
	Foot	19 (28.79)	[39,40,46,48,55,57,59,62,72-74,87,88,91,96,100-103]
	Ankle	16 (24.24)	[38,39,41,45,51,52,61,64,66,70,76,83,93,98,99,103]
	Waist	13 (19.70)	[44,47,52,54,57,65,69,71,86,88,89,97,102]
	Trunk	12 (18.18)	[45,66,74,75,78,81,84,87,88,92,98,102]
	Arm	9 (13.64)	[45,74,75,78,81,84,85,97,98]
	Thigh	7 (10.61)	[55,59,68,74,88,95,102]
	Hip	6 (9.09)	[62,74,78,82,93,98]
	Hand	3 (4.55)	[63,77,91]
	Others (Neck, knees, back, or head)	4 (6.06)	[50,51,74,95]
**Compatibility with OS^c,d^**			
	Local logger	16 (24.24)	[38,40,43,44,46,50,54,57,58,60,61,69,73,87,88,93]
	Android	6 (9.09)	[64,65,76,85,90,97]
	iOS	5 (7.58)	[48,51,63,65,90]
	Windows	3 (4.55)	[68,83,84]
	Not applicable	7 (10.61)	[39,52,67,71,94,95,103]
	Not reported	31 (46.97)	[41,42,45,47,49,53,55,56,59,62,66,70,72,74,75,77-82,86,89,91,92,96,98-102]
**Gateway^e^**			
	PC^f^	10 (15.15)	[52,68,73,74,76,77,80,86,90,102]
	Smartphone	6 (9.09)	[39,48,64,76,85,90]
	Tablet	3 (4.55)	[51,80,92]
	IoT^g^ Gateway	2 (3.03)	[82,103]
	Not reported	48 (72.73)	[38,40-47,49,50,53-63,65-67,69-72,75,78,79,81,83,84,87-89,91,93-101]
**Host^h^**			
	PC^f^	35 (53.03)	[38,41,43,44,52,54-59,61,66,68,69,73-77,80,82-84,86-89,91,93,94,96,98,102,103]
	Server	6 (9.09)	[39,48,58,71,90,97]
	Smartphone	5 (7.58)	[48,64,65,76,85]
	On-device	4 (6.06)	[40,60,65,67]
	Tablet	3 (4.55)	[51,80,92]
	Not reported	18 (27.27)	[42,45-47,49,50,53,62,63,70,72,78,79,81,95,99-101]
**Mode of data transfer^i^**			
	Bluetooth	15 (22.73)	[39,40,48,51,52,64,65,68,76,80,84,86,88,89,102]
	Internet	8 (12.12)	[39,48,58,71,83,85,90,103]
	Removable media	6 (9.09)	[38,40,43,48,61,69]
	Wired	5 (7.58)	[40,44,73,77,103]
	Not reported	39 (59.09)	[41,42,45-47,49,50,53-57,59,60,62,63,66,67,70,72,74,75,78,79,81,82,84,87,91-101]
**Sensors in the wearables^j^**			
	Accelerometer	61 (92.42)	[38-54,57-59,61-99,102,103]
	Gyroscope	50 (75.76)	[38-43,45-49,51,52,54-57,59-64,66-74,76,78,80,81,83,84,86-89,92-94,96,98,99,102,103]
	Magnetometer	11 (16.67)	[45,52,53,66,71,76,78,83,86,87,94]
	Pressure sensor	9 (13.64)	[39,40,46,55,57,59,77,100,101]
	sEMG^k^ sensor	3 (4.55)	[40,42,78]
	Flex sensor	2 (3.03)	[40,77]
**Measured biosignals^l^**			
	Acceleration	61 (92.42)	[38-54,57-59,61-99,102,103]
	Angular velocity	50 (75.76)	[38-43,45-49,51,52,54-57,59-64,66-74,76,78,80,81,83,84,86-89,92-94,96,98,99,102,103]
	Magnetic field signals	11 (16.67)	[45,52,53,66,71,76,78,83,86,87,94]
	Pressure/mechanical signals	9 (13.64)	[39,40,46,55,57,59,77,100,101]
	EMG^m^ signals	3 (4.55)	[40,42,78]
	Bending/Flex sensing	2 (3.03)	[40,77]
**Sensing type**			
	Passive	52 (78.79)	[38,40,41,44-60,62,65,71-73,75-79,82-103]
	Active	14 (21.21)	[39,42,43,61,63,64,66-70,74,80,81]
**Application scenario**			
	Clinical	31 (46.97)	[38,48,51,54,55,57,59-61,63,66-68,74,76-78,80,81,83,84,86,88,89,92-96,98,99]
	Home	12 (18.18)	[44,47,58,65,71,72,79,82,97,100-102]
	Clinical and Home	23 (34.85)	[39-43,45,46,49,50,52,53,56,62,64,69,70,73,75,85,87,90,91,103]
**Duration of monitoring/intervention**			
	Single session	32 (48.48)	[38,40-44,46,48-50,52,55,64,67-69,73,74,76,77,79,83-85,89-91,96,99-102]
	Multiple sessions	20 (30.30)	[47,51,53,54,56,57,60,61,63,66,70,75,80,92-95,97,98,103]
	Long-term monitoring	9 (13.64)	[39,45,58,62,65,71,72,81,86]
	Not reported	5 (7.58)	[59,78,82,87,88]

^a^WD: wearable device.

^b^The number of studies does not add up, as several studies have used >1 wearable device.

^c^OS: operating system.

^d^The number of studies does not add up, as several studies have used >1 wearable device, and many wearable devices are compatible with >1 operating system.

^e^The number of studies does not add up, as several studies used >1 wearable device, and many wearable devices used >1 gateway.

^f^PC: personal computer.

^g^IoT: Internet of Things.

^h^The number of studies does not add up, as several studies used >1 wearable device, and many wearable devices used >1 host.

^i^The number of studies does not add up, as several studies used >1 wearable device, and many wearable devices used >1 mode of data transfer.

^j^The number of studies does not add up, as several studies used >1 wearable device, and most wearable devices have >1 sensor.

^k^sEMG: surface electromyography.

^l^The number of studies does not add up, as several studies used >1 wearable device, and most wearable devices assess >1 biosignal.

^m^EMG: electromyography.

With regard to sensor configuration, devices integrating multiple sensors predominated, accounting for 75.76% (50/66) of the included studies. The collected biosignals were predominantly inertial signals. Accelerometer data were acquired in the vast majority of studies (61/66, 92.42%), followed by gyroscope data (50/66, 75.76%), whereas magnetic field, pressure, and electromyography signals were used less frequently (Table 2).

With respect to application settings, wearable devices were more frequently deployed in clinical environments (31/66, 46.97%), with an additional 34.85% (23/66) of studies spanning both clinical and home environments, whereas studies conducted exclusively in home settings were relatively uncommon (12/66, 18.18%). Regarding the duration of monitoring or intervention, most studies used either single-session assessments (32/66, 48.48%) or repeated short-term testing designs (20/66, 30.30%), while studies involving long-term continuous monitoring were comparatively limited (9/66, 13.64%). Overall, wearable device research in PD is dominated by sensor module devices, with devices most commonly worn on the lower limbs and wrist. Data collection is primarily passive, and studies largely focus on short-term applications conducted in clinical settings. Additional technical characteristics of the wearable devices are provided in Table 2 and Multimedia Appendix 7 [38-103].

### AI Algorithm Characteristics of Wearable Devices

The AI applications in the included studies were categorized into four purposes: (1) monitoring or assessment (35/66, 53.03%), (2) state recognition or functional screening (17/66, 25.76%), (3) prediction (8/66, 12.12%), and (4) rehabilitation and feedback (6/66, 9.09%; Table 3). Among the studies, 62.12% (41/66) used ML algorithms only, 28.79% (19/66) used DL algorithms only, and 9.09% (6/66) combined ML and DL approaches. These studies used algorithms to address classification problems (59/66, 89.39%), regression problems (15/66, 22.73%), and clustering problems (3/66, 4.55%).

**Table 3:** Features of artificial intelligence (AI) algorithms used in the included Parkinson disease wearable-device studies (N=66).

Features			Values, n (%)		References
**AI category**					
	ML^a^	41 (62.12)		[38,39,43,45,48,50,53-55,57-63,65,66,68,71-77,80-84,86-88,92,96,98,99,101-103]	
	DL^b^	19 (28.79)		[41,42,44,46,49,51,52,56,64,67,69,70,78,79,89-91,94,100]	
	ML and DL	6 (9.09)		[40,47,85,93,95,97]	
**Task type^c^**					
	Classification	59 (89.39)		[39-55,57-60,62-67,69,71,72,74-79,81-103]	
	Regression	15 (22.73)		[38,43-45,53,56,61,68-71,77,80,98,102]	
	Clustering	3 (4.55)		[73,77,98]	
**AI algorithm^d^**					
	Support vector machine	28 (42.42)		[38,40,43,45,47,50,53,57,61,65,66,68,74,76,78,81-85,87,88,92,93,96,101-103]	
	Convolutional neural network	23 (34.85)		[40-42,44,46,47,49,51,52,54,56,67,69,70,78,85,89-91,93,95,97,100]	
	Random forest	19 (28.79)		[39,45,47,53,57,60,63,79,80,82,83,86,87,92,95,97-99,101]	
	Logistic regression	15 (22.73)		[39,43,53,61,62,65,71,78-80,87,88,92,98,101]	
	Decision tree	14 (21.21)		[45,47-50,55,57,59,61,62,76,82,86,88]	
	Long short-term memory	11 (16.67)		[42,47,52,56,58,69,70,85,93,94,100]	
	Multilayer perceptron	8 (12.12)		[40,41,44,77,78,88,92,100]	
	k-nearest neighbors	7 (10.61)		[50,76,83,88,92,93,101]	
	Naive Bayes	5 (7.58)		[43,50,76,88,101]	
	Extreme gradient boosting	4 (6.06)		[49,87,93,102]	
	Gradient boosting	3 (4.55)		[39,57,86]	
	Self-attention mechanism	3 (4.55)		[42,69,85]	
	Hidden Markov model	3 (4.55)		[58,72,73]	
	k-means clustering	3 (4.55)		[75,77,98]	
	Elastic net	3 (4.55)		[80,87,98]	
	AdaBoost	3 (4.55)		[76,92,96]	
	Radial basis function	2 (3.03)		[78,79]	
	Others	12 (18.18)		[43,45,47,64,67,78-80,82,86,97,102]	
**Aim of AI algorithm**					
	Monitoring or assessment	35 (53.03)		[38,39,42-47,50,53,56-59,61,63,65,70-73,79,81,83-85,88,90-93,97-99,102]	
	State recognition or functional screening	17 (25.76)		[41,49,51,54,62,66,74-78,82,89,94,100,101,103]	
	Prediction	8 (12.12)		[52,55,64,68,69,80,87,95]	
	Rehabilitation and feedback	6 (9.09)		[40,48,60,67,86,96]	
**Validation approach^e^**					
	Leave-one-out cross-validation	37 (56.06)		[39,41,42,45-47,49,50,53-55,57-63,65-68,70,73,74,76,78,79,81,83,86,88,90,91,93,102,103]	
	k-fold cross-validation	27 (40.91)		[38-41,47,49-52,54,57,69,72,76,77,84,85,87-92,99,101,103]	
	Hold-out validation	18 (27.27)		[40,44,48,49,56,61,64,66,76,78,80,82,88,92,94,100-102]	
	External validation	4 (6.06)		[43,60,69,71]	
	Not reported	5 (7.58)		[75,95-98]	
**Performance measures^f^**					
	Sensitivity	43 (65.15)		[39-42,46,47,49-55,57-60,62,64-67,69,72,73,75-79,82-85,87,90,94-97,99,101,103]	
	Accuracy	41 (62.12)		[39-43,45,46,48,50-55,57,58,62,65,67,69,74-77,82-87,89-92,95-98,100-102]	
	Specificity	27 (40.91)		[46,47,49,50,52-55,59,60,62,64-67,69,76,78,79,82,83,94-96,99,101,103]	
	*F*_1_-score	27 (40.91)		[39-41,49,51-55,57,67,73,75-78,82,84,85,87,92,93,95,99,101-103]	
	Area under the curve	25 (37.88)		[39-41,47,52,55,57,58,62-65,69,74,78,79,82,86-88,92,94,96,98,101]	
	Precision	24 (36.36)		[39-42,46,51,53,54,58,59,62,65-67,75-78,84,85,87,92,95,97]	
	Correlation coefficient	14 (21.21)		[38,44,53,56,58,61,68,70,71,79-81,88,102]	
	Receiver operating characteristic	7 (10.61)		[60,63,66,74,89,94,98]	
	Mean absolute error	7 (10.61)		[43,44,56,68,70,98,102]	
	Root-mean-square error	6 (9.09)		[38,53,61,68,80,102]	
	Intraclass correlation coefficient	4 (6.06)		[38,54,77,93]	
	Cohen κ	3 (3.03)		[77,95,102]	
	Equal error rate	2 (3.03)		[47,52]	
	G-mean	2 (3.03)		[51,52]	
	Negative predictive value	2 (3.03)		[52,65]	
	Coefficient of determination	2 (3.03)		[70,98]	
	Others	9 (13.64)		[38,52,54,59,66,67,82,88,99]	

^a^ML: machine learning.

^b^DL: deep learning.

^c^The number of studies does not add up, as many studies have used >1 task type.

^d^The number of studies does not add up, as many studies have used >1 AI algorithm.

^e^The number of studies does not add up, as many studies have used >1 validation approach.

^f^The number of studies does not add up, as most studies used >1 performance measure.

A total of 30 distinct AI algorithms were identified across the included studies. The most frequently used were support vector machines (SVMs; 28/66, 42.42%), followed by convolutional neural networks (CNNs; 23/66, 34.85%), random forests (19/66, 28.79%), logistic regression (15/66, 22.73%), decision trees (14/66, 21.21%), long short-term memory (LSTM) networks (11/66, 16.67%), and multilayer perceptrons (8/66, 12.12%; Table 3).

**Figure 4:**
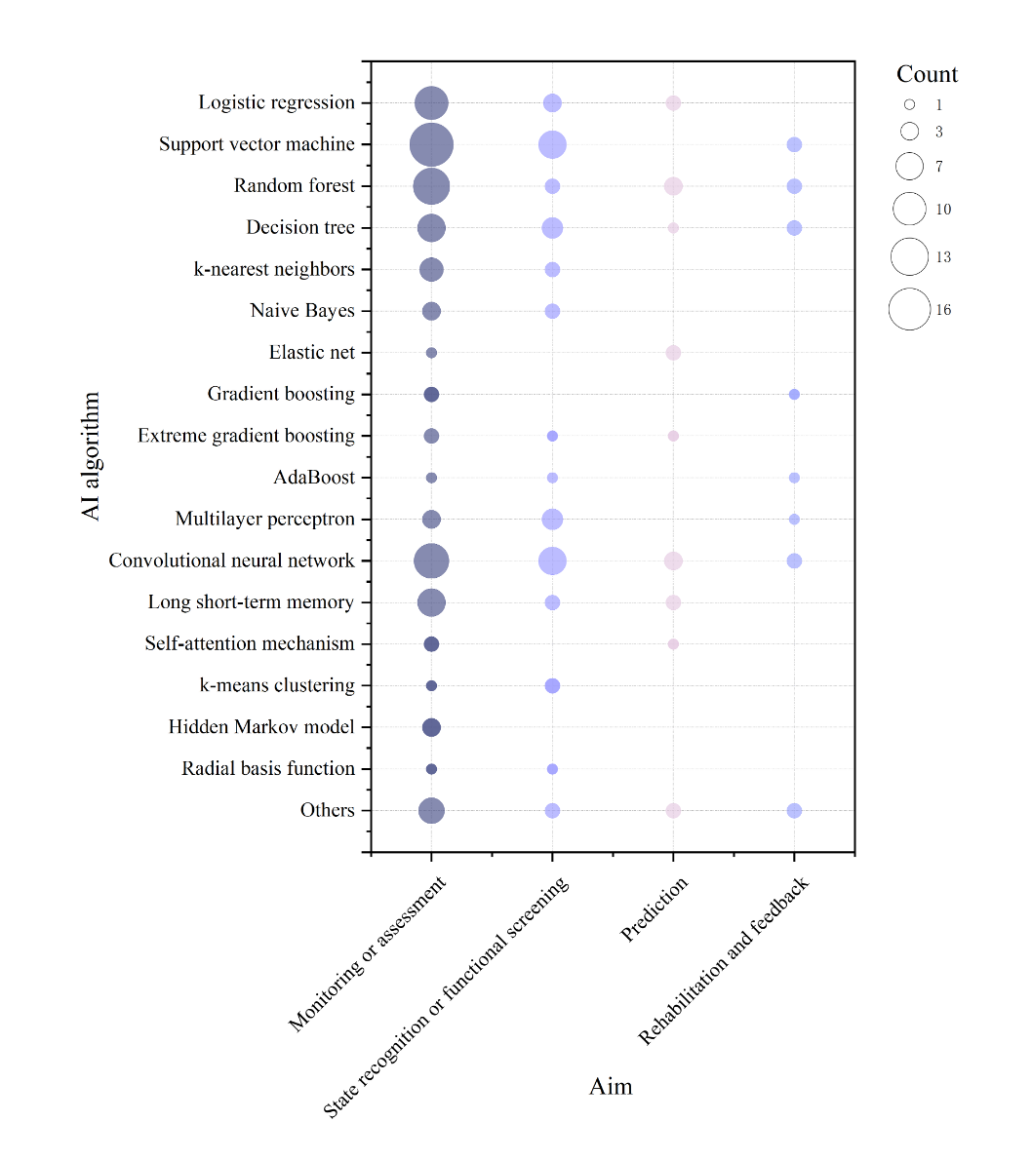
Distribution of artificial intelligence (AI) algorithms and application objectives in the included Parkinson disease wearable-device studies.

As shown in Figure 4, SVMs and CNNs were predominantly applied in the “monitoring or assessment” and “state recognition or functional screening” categories. Random forests, decision trees, and logistic regression models were distributed across application purposes but were mainly used for monitoring- or assessment-related tasks. Temporal and probabilistic models, such as LSTM networks and hidden Markov models, were more often observed in state recognition or functional screening tasks, although their overall adoption remained limited. In contrast, algorithmic applications in the “prediction” and “rehabilitation or feedback” categories were comparatively sparse, indicating that prospective and closed-loop applications remain at an early stage of development.

A total of 4 different model validation approaches were used across the included studies, with approximately 31.82% (21/66) using more than 1 method (Table 3). Leave-one-out cross-validation was the most frequently used approach (37/66, 56.06%), followed by k-fold cross-validation (27/66, 40.91%), hold-out validation (18/66, 27.27%), and external validation (4/66, 6.06%). A total of 25 performance metrics were used to evaluate model performance. The most commonly reported metrics were sensitivity (43/66, 65.15%), accuracy (41/66, 62.12%), specificity (27/66, 40.91%), *F*_1_-score (27/66, 40.91%), area under the curve (25/66, 37.88%), precision (24/66, 36.36%), and correlation coefficient (14/66, 21.21%). Detailed characteristics of the AI algorithms applied in each study are provided in Multimedia Appendix 8 [38-103].

### Challenges and Opportunities of Wearable Devices

**Figure 5:**
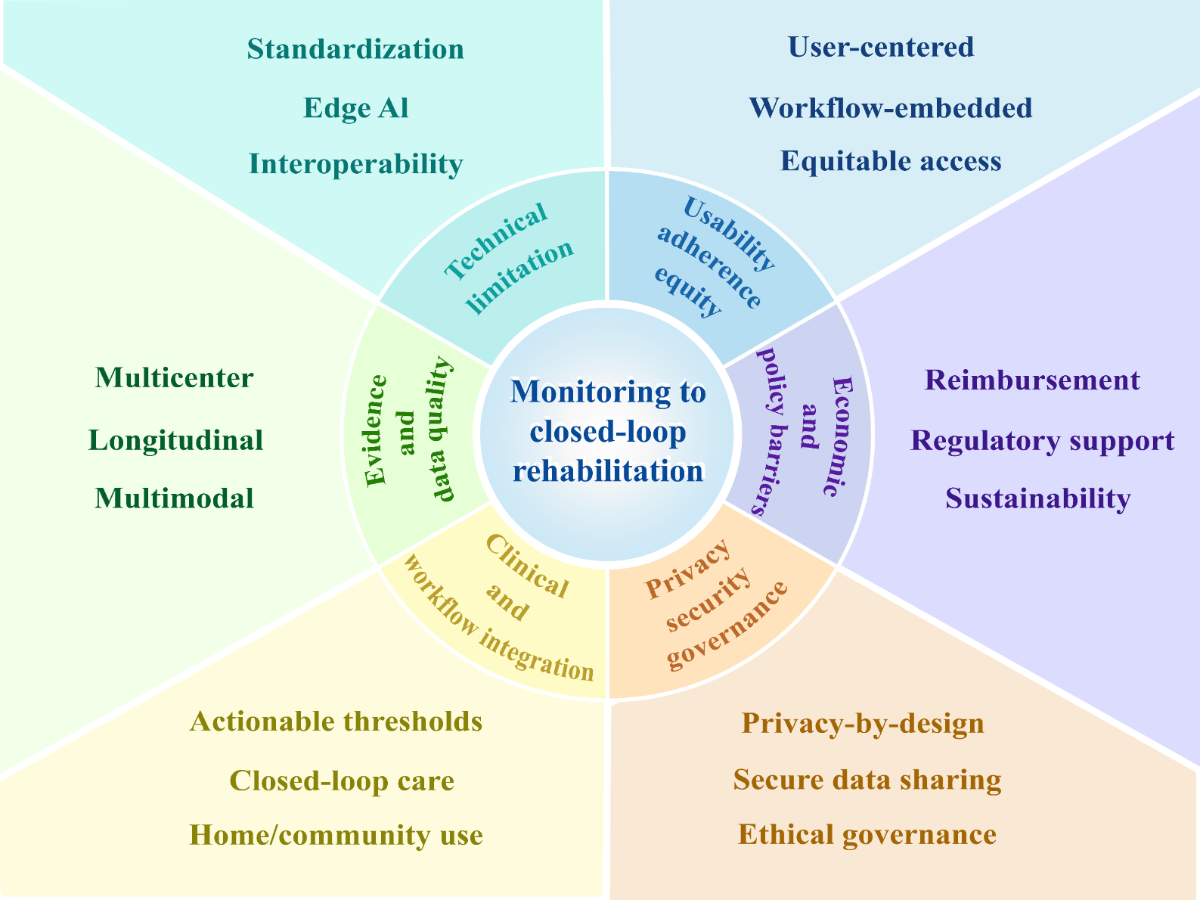
From monitoring to closed-loop rehabilitation: key opportunity domains for artificial intelligence (AI)–enabled wearable devices in Parkinson disease.

Based on a synthesis of the included evidence, we developed a conceptually integrated “challenges and opportunities” framework grounded in the evidence base. This framework categorizes the challenges and opportunities of AI-enabled wearable devices in clinical applications into six dimensions: (1) evidence and data quality; (2) technical limitations; (3) usability, adherence, and equity; (4) economic and policy barriers; (5) privacy, security, and data governance; and (6) clinical translation and workflow integration. The framework is intended to inform future research and practice rather than to represent an empirically validated implementation pathway, as illustrated in Figure 5 and detailed in Multimedia Appendix 9.

## Discussion

### Principal Findings

#### Overview

We synthesized the evidence on AI-enabled wearable devices for PD rehabilitation and motor function assessment, focusing on device types, monitoring indicators, algorithmic approaches, and application characteristics. Overall, the available evidence remains predominantly monitoring- and assessment-oriented, with relatively limited rehabilitation interventions and workflow-integrated applications. Key translational gaps persist, including limited evidence from real-world home/community settings, scarce external validation, underreporting of model calibration and clinical use, and underrepresentation of diverse populations.

Most prior reviews were published between 2020 and 2023 [104-106], and may not fully capture more recent developments, including the wider adoption of remote monitoring devices [107,108], rapid advances in commercial multisensor systems [109], and the evolution of AI models [110]. In contrast to earlier reviews that primarily focused on diagnosis or device technical performance, the present review adopts a rehabilitation- and nursing-oriented perspective and foregrounds evidence relevant to real-world implementation and nurse-led translation. Building on these findings, we propose a conceptual closed-loop framework linking monitoring, assessment, intervention, feedback, and reevaluation, which can be tested and refined in future research.

#### Study Characteristics

This review covered the 2020-2025 publication window and found that the volume of studies remained consistently high in recent years, suggesting that the field has entered a relatively stable phase of development rather than a period of rapid expansion, consistent with previous reviews [106]. Nevertheless, notable limitations in methodological rigor and sampling design persist: (1) sample sizes varied substantially, with mean values often influenced by outliers [48,80]; (2) sampling was typically based on single-center convenience cohorts [41,64,96]; and (3) subgroup analyses by sex, age, or special populations (such as early-onset or comorbid PD) were largely absent [41,83,84,96]. Many studies enrolled only people with PD, with relatively few including healthy control groups. Outcome reporting tended to prioritize discrimination metrics (eg, accuracy), while model calibration, uncertainty quantification, and clinically actionable thresholding or decision-curve analysis were rarely reported [57,63,64,72,94-96]. In addition, research has been concentrated in Europe and North America (Table 1), with comparatively limited evidence from low- and middle-income countries and insufficient attention to home/community settings, thereby constraining generalizability and real-world applicability. Overall, persistent methodological limitations—including small, single-center samples, predominantly internal validation, and limited evidence from home/community settings—suggest that growth in study quantity has outpaced advances in translational value.

#### Application Characteristics and Signal Focus

In terms of application characteristics, AI-enabled wearable devices in PD show a marked imbalance between assessment-oriented and rehabilitation-oriented work, consistent with prior systematic reviews describing the field as “detection-heavy but prediction- and intervention-light” [104-106]. Although several representative studies have demonstrated the feasibility of continuous sensor-based quantification of tremor and bradykinesia [53], objective quantification of medication response [38], and sensor-based early detection of freezing of gait [54,55], most remain at a proof-of-concept stage, with limited evidence for real-world, workflow-integrated, or actionable use in routine care [104,105]. This disparity likely reflects inherent differences in methodological complexity. Assessment-focused studies typically rely on passive data capture and brief, structured tasks that are easier to implement in controlled settings, whereas rehabilitation studies require longer follow-up, personalized feedback, adherence management, and integration with clinical workflows—substantially raising both technical and methodological demands [13,111]. Moreover, most studies adopt motion capture–oriented wearable devices centered on inertial and/or pressure signals, with sensors preferentially placed on the lower limbs or the wrist. This design choice reduces data-collection burden and improves task reproducibility, thereby reinforcing assessment paradigms built around controlled tasks; however, it also structurally steers research toward motor outcomes (eg, gait, freezing of gait, tremor, and bradykinesia), making sleep, mood, and cognition more difficult to capture continuously and comprehensively [38,46,53]. Meanwhile, many applications still rely on short, structured assessments, which further constrain the continuous capture of nonmotor domains in real-world contexts [16,19]. Taken together, the evidence continues to prioritize motor-domain tasks, with insufficient coverage of nonmotor symptoms and their continuous assessment in real-world contexts. As a result, current applications fall short of meeting the comprehensive needs of long-term chronic disease management.

#### AI and Algorithmic Characteristics

In our review, SVMs and CNNs are the most frequently used approaches, while decision trees and logistic regression are commonly used as interpretable baseline models. Currently, the field reflects a pattern of “traditional ML dominance, parallel advances in convolutional DL, and early-stage development of sequential models and emerging paradigms” [38,40,50,57,68,74], which aligns with the ML/DL distribution observed in our results. This distribution may be attributable to differences in how algorithms adapt to data scale and structure. SVMs tend to provide more stable performance in small-sample, feature-engineered wearable data settings, whereas CNNs are better suited to end-to-end feature learning from high-frequency inertial signals, thereby reducing reliance on manual feature engineering [112,113]. Sequential approaches (eg, LSTM networks and a small number of HMMs) have been explored for gait-phase and state-recognition tasks, but remain relatively uncommon. This suggests that temporal dependencies and state-transition dynamics are still underutilized [36,52,72,73]. Consistent with these constraints, validation remains largely internal (eg, cross-validation), while independent external validation is rare, limiting confidence in generalizability [105]. More complex architectures (eg, self-attention mechanisms and extreme gradient boosting) also appear underused, potentially reflecting feasibility constraints for on-device or real-world deployment [39,75,80,92-96,98].

In summary, AI-enabled wearable devices for PD rehabilitation and assessment remain characterized by a strong emphasis on monitoring, with comparatively weak integration into closed-loop systems. From the perspective of the *JMIR mHealth and uHealth* taxonomy, most devices included in this review are better characterized as mobile health tools, primarily focused on individual-level monitoring and assessment [114]. Although these functions provide valuable objective data, there is still insufficient evidence that they have been systematically embedded into routine nurse-led rehabilitation workflows or broader health care delivery systems as mature eHealth solutions. Building on these findings, future research should move beyond stand-alone monitoring applications toward workflow-integrated eHealth systems with decision-support functions that can be tested in nurse-led rehabilitation pathways.

### Practical Recommendations

#### Implications for Clinical and Nursing Practice

In both clinical and home-based rehabilitation contexts, nurses increasingly require continuous and objective patient information to enhance the precision of assessment and monitoring, and strengthen patient engagement in the rehabilitation process [115]. AI-enabled wearable devices represent a promising approach to addressing this need. Within clinical rehabilitation settings, continuous monitoring of gait, tremor, sleep, and activity levels can augment routine nursing surveillance, facilitate earlier detection of functional deterioration, and support timely intervention or prioritization—moving beyond the inherently fragmented nature of episodic in-clinic observations [25,115]. In remote or home-based rehabilitation, the continuous and objective data generated by wearables can be used to evaluate therapeutic response and adherence, while personalized feedback, symptom prompts, medication-taking support, and nurse-led follow-up via telerehabilitation platforms promote patient self-management [26,115]. Specifically, when algorithmic detection identifies abnormal changes in patients’ daily activity levels or tremor severity that exceed an individualized threshold, an alert can be triggered on the nursing interface. This may prompt nurses to rapidly assess symptom changes, medication adherence, and potential adverse effects and, as appropriate, arrange earlier review, reinforce medication and safety guidance, or adjust follow-up plans [97,115]. Likewise, when long-term monitoring trend reports indicate sustained deterioration in motor function–related indicators or marked fluctuations in motor symptoms, these data can provide objective evidence for the rehabilitation team to deliver targeted health education, adjust the frequency of follow-up, and, when needed, coordinate involvement of rehabilitation therapists or a multidisciplinary team [56,89,99]. These functions align closely with core nursing responsibilities in patient education, symptom monitoring, and long-term chronic disease management, and may help reinforce the full nursing care cycle of assessment, diagnosis, planning, implementation, and evaluation [116].

#### Priorities for Future Research and Translation

To advance the field, future research may consider five priority areas. (1) Strengthening the evidence base through multicenter, long-term real-world studies with preregistration, appropriate sample size estimation, and attrition management, alongside reporting of external validation, model calibration, uncertainty, and clinical use [117-120]. (2) Enhancing model feasibility through multimodal signal fusion and data-efficient learning strategies to support practical real-world deployment [104]. (3) Exploring conceptual closed-loop rehabilitation frameworks that integrate assessment and intervention, while examining the feasibility of actionable thresholds and their potential integration into routine clinical workflows [120]. (4) Improving device comfort and usability to balance patient adherence with caregiver and clinician workload, address the digital divide, and enhance accessibility [119,120]. (5) Strengthening privacy and data security frameworks, alongside improving insurance reimbursement mechanisms and regulatory support, to promote responsible cross-institutional data sharing [121,122], including explicitly addressing equity, responsible data use, and related ethical considerations in real-world nursing and rehabilitation contexts.

### Limitations

This study has several limitations. First, no prospective protocol was registered; however, this is an acceptable practice for scoping reviews, and we clarify this point to enhance methodological transparency. Second, although a systematic search and screening process was conducted, the scope of databases, search strategies, and inclusion criteria may have led to the omission of eligible studies, posing a risk of incompleteness or potential bias. In addition, most included studies had small sample sizes, single-center designs, short follow-up durations, and limited external validation, alongside substantial heterogeneity across study populations, task paradigms, signal sources, algorithmic approaches, and outcome measures. This heterogeneity may limit cross-study comparability and the generalizability of findings. Given the predominantly exploratory and proof-of-concept nature of the included studies, the closed-loop rehabilitation framework and related recommendations proposed in this review should be interpreted as conceptual and practice-informing rather than empirically validated implementation pathways. Finally, the search was restricted to English and Chinese peer-reviewed publications, with limited inclusion of gray literature or other languages, which may introduce time-lag, linguistic, and publication biases. In particular, studies reporting null or negative findings, early-stage failures, or limited model performance may be less likely to be published and therefore underrepresented, potentially leading to an overestimation of the maturity, effectiveness, and translational readiness of AI-enabled wearable technologies.

### Conclusions

This study systematically summarized recent evidence on AI-enabled wearable devices for motor function assessment and rehabilitation in people with PD. By combining a scoping review with an evidence map and adopting a rehabilitation-oriented perspective, we synthesized evidence across multiple dimensions, including application aims, sensing modalities, AI methods, and validation practices, thereby identifying key translational gaps between proof-of-concept studies and real-world rehabilitation workflows. Compared with previous reviews that primarily focused on monitoring functions or device performance, this study places greater emphasis on rehabilitation applications and nurse-led translation into practice, and further clarifies the key actionable gaps that limit real-world implementation. On this basis, a conceptually integrated “challenges and opportunities” framework is proposed to inform the design, evaluation, and reporting of devices and algorithms, and to highlight considerations for developing workflow-integrated, decision-support wearable systems. From a real-world perspective, these findings may support continuity of rehabilitation across clinical, home, and community settings by enabling nurses to deliver continuous monitoring, personalized follow-up, and timely intervention, ultimately improving the efficiency and accessibility of PD rehabilitation management.

## Data Availability

This scoping review did not generate any primary data. All data extracted and synthesized in this review were obtained from previously published studies, which are publicly available. No additional datasets were created or analyzed.

## Appendix

Multimedia Appendix 1 PRISMA-ScR checklist.

Multimedia Appendix 2 Eligibility criteria.

Multimedia Appendix 3 Search strategy.

Multimedia Appendix 4 Extracted data form.

Multimedia Appendix 5 Quality assessment of the included studies.

Multimedia Appendix 6 Detailed characteristics of included studies using artificial intelligence–enabled wearable devices in Parkinson disease research.

Multimedia Appendix 7 Detailed characteristics of artificial intelligence–enabled wearable devices used in the included Parkinson disease studies.

Multimedia Appendix 8 Detailed characteristics of artificial intelligence algorithms used in the included wearable device studies for Parkinson disease.

Multimedia Appendix 9 Challenges and opportunities of artificial intelligence–enabled wearable devices in Parkinson disease research.

## Abbreviations

AI: artificial intelligence
CNKI: China National Knowledge Infrastructure
CNN: convolutional neural network
DL: deep learning
LSTM: long short-term memory
MeSH: Medical Subject Headings
ML: machine learning
PD: Parkinson disease
PRISMA: Preferred Reporting Items for Systematic Reviews and Meta-Analyses
PRISMA-S: Preferred Reporting Items for Systematic Reviews and Meta-Analyses literature search extension
PRISMA-ScR: Preferred Reporting Items for Systematic Reviews and Meta-Analyses extension for Scoping Reviews
SVM: support vector machine
