# Correction: AI-Enabled Wearables for Motor Function Assessment and Rehabilitation in Parkinson Disease: Scoping Review

**DOI:** 10.2196/97418

**Published:** 2026-04-21

**Authors:** Shengting Li, Siqi Chen, Xiaosong Yu, Huixiang Shang, Tingting Tu, Mingtao Quan

**Affiliations:** 1 Department of Nursing Affiliated Hospital of Zunyi Medical College Zunyi, Guizhou China; 2 School of Nursing Zunyi Medical University Zunyi, Guizhou China

In “AI-Enabled Wearables for Motor Function Assessment and Rehabilitation in Parkinson Disease: Scoping Review,” the authors requested a correction to the author affiliations.

In the published article, the author affiliations were presented as:

^1^School of Nursing, Zunyi Medical University, Zunyi, China

^2^Affiliated Hospital of Zunyi Medical College, Zunyi, China

These have been revised to the following:

^1^Department of Nursing, Affiliated Hospital of Zunyi Medical College, Zunyi, Guizhou, China

^2^School of Nursing, Zunyi Medical University, Zunyi, Guizhou, China

This correction does not affect the authorship, author order, or the scientific content of the article.

The correction will appear in the online version of the paper on the JMIR Publications website, together with the publication of this correction notice. Because this was made after submission to PubMed, PubMed Central, and other full-text repositories, the corrected article has also been resubmitted to those repositories.
