# Supplementary material for: AI-Enabled Wearables for Motor Function Assessment and Rehabilitation in Parkinson Disease: Scoping Review
Source: J Med Internet Res. 2026 Feb 26;28:e85596. doi: 10.2196/85596 (PMC12982951; doi:10.2196/85596)
Supplement: Multimedia Appendix 2 [file jmir_v28i1e85596_app2.docx]

**Appendix 2. Eligibility criteria.**

|  | **Inclusion** | **Exclusion** |
| --- | --- | --- |
| **Population** | Patients diagnosed with Parkinson’s disease (PD), with no restriction on age, gender, or disease stage. Studies including mixed populations will be eligible only if PD data can be extracted separately. | Studies without PD patients or where PD-specific data cannot be separated. |
| **Concept** | Studies using non-invasive, body-worn wearable or wearable-type mobile devices combined with AI techniques (e.g., machine learning, deep learning). Devices include smartwatches, wristbands, smart insoles, smart clothing, exoskeletons, wearable sensor systems, electronic textiles, and wearable-type mobile terminals. Applications must be related to rehabilitation or motor function assessment, such as:   - Motor function assessment (gait, posture, tremor, joint mobility, etc.) - Rehabilitation training feedback and efficacy evaluation - Monitoring disease progression or changes in motor function - Optimization of personalized rehabilitation programs or predictive modeling. Optimization of personalized rehabilitation programs or predictive modeling directly related to rehabilitation or functional assessment. - State recognition or functional screening | Studies not applying AI methods (only traditional statistics/thresholds). Studies using non-wearable devices, handheld-only devices (e.g., smartphones), near-body but non-wearable devices, implanted devices, or devices requiring wired connections to external systems. |
| **Context** | Rehabilitation medicine and clinical nursing practice, including long-term follow-up and home or community-based rehabilitation | Studies limited to disease diagnosis or risk prediction settings without relevance to rehabilitation. |
| **Type of source** | Original research articles, theses/dissertations, and conference papers, published on or after 1 January 2020 to reflect recent progress. Both Chinese and English language studies will be included. | Reviews, case reports, preprints, study protocols, conference abstracts, posters, editorials, and commentaries. |
