# Supplementary material for: AI-Enabled Wearables for Motor Function Assessment and Rehabilitation in Parkinson Disease: Scoping Review
Source: J Med Internet Res. 2026 Feb 26;28:e85596. doi: 10.2196/85596 (PMC12982951; doi:10.2196/85596)
Supplement: Multimedia Appendix 3 [file jmir_v28i1e85596_app3.docx]

**Appendix 3. Search Strategy**

**Pubmed：**

| **Line** | **Searches** | **NO.** |
| --- | --- | --- |
| #1 | "Wearable Electronic Devices"[MeSH Terms] OR "Exoskeleton Device"[MeSH Terms] OR ("Robotics"[MeSH Terms] AND "wearable"[Title/Abstract]) OR "wearable"[Title/Abstract] OR "wearable device*"[Title/Abstract] OR "wearable sensor*"[Title/Abstract] OR "smart wearable*"[Title/Abstract] OR "smart clothing"[Title/Abstract] OR "smart garment*"[Title/Abstract] OR "e textile*"[Title/Abstract] OR "electronic textile*"[Title/Abstract] OR "smart textile*"[Title/Abstract] OR "exoskeleton*"[Title/Abstract] OR "wearable robot*"[Title/Abstract] OR "wearable orthosis"[Title/Abstract] OR "smartwatch*"[Title/Abstract] OR "smart watch"[Title/Abstract] OR "fitness tracker*"[Title/Abstract] OR "activity tracker*"[Title/Abstract] OR "pedometer*"[Title/Abstract] OR "smart shoe*"[Title/Abstract] OR "smart insole*"[Title/Abstract] | 65,192 |
| #2 | "Rehabilitation"[MeSH Terms] OR "Motor Activity"[MeSH Terms] OR "Gait Analysis"[MeSH Terms] OR "rehabilitation"[Title/Abstract] OR "motor recovery"[Title/Abstract] OR "motor rehabilitation"[Title/Abstract] OR "gait analysis"[Title/Abstract] OR "balance training"[Title/Abstract] OR "movement analysis"[Title/Abstract] OR "rehabilitation training"[Title/Abstract] OR "motor function assessment"[Title/Abstract] OR "motor function evaluation"[Title/Abstract] OR "activity monitoring"[Title/Abstract] OR "physiologic monitoring"[Title/Abstract] OR "recovery monitoring"[Title/Abstract] OR "therapy feedback"[Title/Abstract] OR "postural control"[Title/Abstract] OR "tremor assessment"[Title/Abstract] OR "bradykinesia assessment"[Title/Abstract] | 913,506 |
| #3 | "Parkinson Disease"[MeSH Terms] OR "parkinson*"[Title/Abstract] | 179,835 |
| #4 | "Artificial Intelligence"[MeSH Terms] OR "Machine Learning"[MeSH Terms] OR "Deep Learning"[MeSH Terms] OR "artificial intelligence"[Title/Abstract] OR "machine learning"[Title/Abstract] OR "deep learning"[Title/Abstract] OR "neural network*"[Title/Abstract] OR "algorithm*"[Title/Abstract] OR "supervised learning"[Title/Abstract] OR "unsupervised learning"[Title/Abstract] OR "reinforcement learning"[Title/Abstract] | 835,881 |
| #5 | #1 AND #2 AND #3 AND #4 | 178 |

**Embase：**

| **Line** | **Searches** | **NO.** |
| --- | --- | --- |
| #1 | ('wearable electronic device'/exp OR 'exoskeleton device'/exp OR ('robotics'/exp AND wearable:ti,ab) OR wearable:ti,ab OR 'wearable device*':ti,ab OR 'wearable sensor*':ti,ab OR 'smart wearable*':ti,ab OR 'smart clothing':ti,ab OR 'smart garment*':ti,ab OR 'e textile*':ti,ab OR 'electronic textile*':ti,ab OR 'smart textile*':ti,ab OR exoskeleton*:ti,ab OR 'wearable robot*':ti,ab OR 'wearable orthosis':ti,ab OR smartwatch*:ti,ab OR 'smart watch':ti,ab OR 'fitness tracker*':ti,ab OR 'activity tracker*':ti,ab OR pedometer*:ti,ab OR 'smart shoe*':ti,ab OR 'smart insole*':ti,ab) | 71,346 |
| #2 | ('rehabilitation'/exp OR 'motor activity'/exp OR 'gait analysis'/exp OR rehabilitation:ti,ab OR 'motor recovery':ti,ab OR 'motor rehabilitation':ti,ab OR 'gait analysis':ti,ab OR 'balance training':ti,ab OR 'movement analysis':ti,ab OR 'rehabilitation training':ti,ab OR 'motor function assessment':ti,ab OR 'motor function evaluation':ti,ab OR 'activity monitoring':ti,ab OR 'physiologic monitoring':ti,ab OR 'recovery monitoring':ti,ab OR 'therapy feedback':ti,ab OR 'postural control':ti,ab OR 'tremor assessment':ti,ab OR 'bradykinesia assessment':ti,ab) | 1,520,311 |
| #3 | ('parkinson disease'/exp OR parkinson*:ti,ab) | 291,301 |
| #4 | ('artificial intelligence'/exp OR 'machine learning'/exp OR 'deep learning'/exp OR 'artificial intelligence':ti,ab OR 'machine learning':ti,ab OR 'deep learning':ti,ab OR 'neural network*':ti,ab OR algorithm*:ti,ab OR 'supervised learning':ti,ab OR 'unsupervised learning':ti,ab OR 'reinforcement learning':ti,ab) | 1,219,928 |
| #5 | #1 AND #2 AND #3 AND #4 | 229 |

**Web of Science**

| **Line** | **Searches** | **NO.** |
| --- | --- | --- |
| #1 | TS=("wearable electronic device*" OR "exoskeleton device*" OR ("robotics" AND wearable)  OR wearable OR "wearable device*" OR "wearable sensor*" OR "smart wearable*"  OR "smart clothing" OR "smart garment*" OR "e textile*" OR "electronic textile*"  OR "smart textile*" OR exoskeleton* OR "wearable robot*" OR "wearable orthosis"  OR smartwatch* OR "smart watch" OR "fitness tracker*" OR "activity tracker*"  OR pedometer* OR "smart shoe*" OR "smart insole*") | 138,230 |
| #2 | TS=("rehabilitation" OR "motor activity" OR "gait analysis" OR neurorehabilitation  OR "neurological rehabilitation" OR "motor recovery" OR "motor rehabilitation"  OR "balance training" OR "upper limb function" OR "movement analysis" OR "rehabilitation training" OR "motor function assessment" OR "motor function evaluation" OR "activity monitoring" OR "physiologic monitoring" OR "recovery monitoring" OR "therapy feedback" OR "postural control" OR "tremor assessment" OR "bradykinesia assessment") | 597,611 |
| #3 | TS=(parkinson* OR "parkinson's disease") | 283,135 |
| #4 | TS=("artificial intelligence" OR "machine learning" OR "deep learning"  OR "neural network*" OR algorithm* OR "supervised learning"  OR "unsupervised learning" OR "reinforcement learning") | 4,350,007 |
| #5 | #1 AND #2 AND #3 AND #4 | 316 |

**Cochrane Library**

| **Line** | **Searches** | **NO.** |
| --- | --- | --- |
| #1 | MeSH descriptor: [Wearable Electronic Devices] explode all trees | 1,193 |
| #2 | (wearable OR wearable NEXT device* OR wearable NEXT sensor* OR smart NEXT wearable*  OR smart NEXT clothing OR smart NEXT garment* OR e NEXT textile* OR electronic NEXT textile*  OR smart NEXT textile* OR exoskeleton* OR wearable NEXT robot* OR wearable NEXT orthosis  OR smartwatch* OR smart NEXT watch OR fitness NEXT tracker* OR activity NEXT tracker*  OR pedometer* OR smart NEXT shoe* OR smart NEXT insole* OR robotics):ti,ab,kw | 9,658 |
| #3 | #1 OR #2 | 10,179 |
| #4 | MeSH descriptor: [Rehabilitation] explode all trees | 58,607 |
| #5 | MeSH descriptor: [Motor Activity] explode all trees | 45,301 |
| #6 | MeSH descriptor: [Gait Analysis] explode all trees | 86 |
| #7 | (neurorehabilitation OR "neurological rehabilitation" OR "motor recovery"  OR "motor rehabilitation" OR "gait analysis" OR "balance training"  OR "upper limb function" OR "movement analysis" OR "rehabilitation training"  OR "motor function assessment" OR "motor function evaluation"  OR "activity monitoring" OR "physiologic monitoring" OR "recovery monitoring"  OR "therapy feedback" OR "postural control" OR "tremor assessment"  OR "bradykinesia assessment"):ti,ab,kw | 14,861 |
| #8 | #4 OR #5 OR #6 OR #7 | 100,279 |
| #9 | MeSH descriptor: [Parkinson Disease] explode all trees | 6,445 |
| #10 | (parkinson*):ti,ab,kw | 15,554 |
| #11 | #9 OR #10 | 15,554 |
| #12 | MeSH descriptor: [Artificial Intelligence] explode all trees | 3,835 |
| #13 | MeSH descriptor: [Machine Learning] explode all trees | 1,246 |
| #14 | MeSH descriptor: [Deep Learning] explode all trees | 401 |
| #15 | ("artificial intelligence" OR "machine learning" OR "deep learning"  OR neural NEXT network* OR algorithm* OR "supervised learning"  OR "unsupervised learning" OR "reinforcement learning"):ti,ab,kw | 26,971 |
| #16 | #12 OR #13 OR #14 OR #15 | 28,378 |
| #17 | #3 AND #8 AND #11 AND #16 | 7 |

**Scopus**

| **Line** | **Searches** | **NO.** |
| --- | --- | --- |
| #1 | TITLE-ABS-KEY("wearable" OR "wearable device*" OR "wearable sensor*" OR "smart wearable*"  OR "smart clothing" OR "smart garment*" OR "e textile*" OR "electronic textile*"  OR "smart textile*" OR exoskeleton* OR "wearable robot*" OR "wearable orthosis"  OR smartwatch* OR "smart watch" OR "fitness tracker*" OR "activity tracker*"  OR pedometer* OR "smart shoe*" OR "smart insole*" OR "inertial sensor*" OR accelerometer*) | 264,611 |
| #2 | TITLE-ABS-KEY("rehabilitation" OR neurorehabilitation OR "neurological rehabilitation"  OR "motor recovery" OR "motor rehabilitation" OR "gait analysis" OR "balance training"  OR "upper limb function" OR "movement analysis" OR "rehabilitation training"  OR "motor function assessment" OR "motor function evaluation" OR "activity monitoring"  OR "physiologic monitoring" OR "recovery monitoring" OR "therapy feedback"  OR "postural control" OR "tremor assessment" OR "bradykinesia assessment"  OR "feedback system*") | 688,489 |
| #3 | TITLE-ABS-KEY(parkinson* OR "parkinson's disease") | 260,363 |
| #4 | TITLE-ABS-KEY("artificial intelligence" OR "machine learning" OR "deep learning"  OR "neural network*" OR algorithm* OR "supervised learning"  OR "unsupervised learning" OR "reinforcement learning") | 6,608,641 |
| #5 | #1 AND #2 AND #3 AND #4 | 247 |

**CINAHL**

| **Line** | **Searches** | **NO.** |
| --- | --- | --- |
| #1 | (MH "Wearable Electronic Devices+") OR (MH "Exoskeletons+") OR (MH "Robotics+") OR  TI(wearable OR "wearable device*" OR "wearable sensor*" OR "smart wearable*" OR "smart clothing" OR "smart garment*" OR "e textile*" OR "electronic textile*" OR "smart textile*" OR exoskeleton* OR "wearable robot*" OR "wearable orthosis" OR smartwatch* OR "smart watch" OR "fitness tracker*" OR "activity tracker*" OR pedometer* OR "smart shoe*" OR "smart insole*") OR  AB(wearable OR "wearable device*" OR "wearable sensor*" OR "smart wearable*" OR "smart clothing" OR "smart garment*" OR "e textile*" OR "electronic textile*" OR "smart textile*" OR exoskeleton* OR "wearable robot*" OR "wearable orthosis" OR smartwatch* OR "smart watch" OR "fitness tracker*" OR "activity tracker*" OR pedometer* OR "smart shoe*" OR "smart insole*") | 183,746 |
| #2 | (MH "Rehabilitation+") OR (MH "Motor Activity+") OR (MH "Gait Analysis+") OR  TI("rehabilitation" OR "motor recovery" OR "motor rehabilitation" OR "gait analysis" OR "balance training" OR "movement analysis" OR "rehabilitation training" OR "motor function assessment" OR "motor function evaluation" OR "activity monitoring" OR "physiologic monitoring" OR "recovery monitoring" OR "therapy feedback" OR "postural control" OR "tremor assessment" OR "bradykinesia assessment") OR  AB("rehabilitation" OR "motor recovery" OR "motor rehabilitation" OR "gait analysis" OR "balance training" OR "movement analysis" OR "rehabilitation training" OR "motor function assessment" OR "motor function evaluation" OR "activity monitoring" OR "physiologic monitoring" OR "recovery monitoring" OR "therapy feedback" OR "postural control" OR "tremor assessment" OR "bradykinesia assessment") | 1,902,275 |
| #3 | (MH "Parkinson Disease+") OR TI(parkinson*) OR AB(parkinson*) | 273,311 |
| #4 | (MH "Artificial Intelligence+") OR (MH "Machine Learning+") OR (MH "Deep Learning+") OR  TI("artificial intelligence" OR "machine learning" OR "deep learning" OR "neural network*" OR algorithm* OR "supervised learning" OR "unsupervised learning" OR "reinforcement learning") OR  AB("artificial intelligence" OR "machine learning" OR "deep learning" OR "neural network*" OR algorithm* OR "supervised learning" OR "unsupervised learning" OR "reinforcement learning") | 1,609,131 |
| #5 | #1 AND #2 AND #3 AND #4 | 125 |

**China National Knowledge Infrastructure**

| **Line** | **Searches** | **NO.** |
| --- | --- | --- |
| #1 | (SU %= '可穿戴' + '智能穿戴' + '穿戴式' + '可穿戴传感器' + '外骨骼' + '智能服装' + '智能纺织品' + '电子纺织品' + '智能手表' + '智能手环' + '追踪器' + '计步器') | 34,795 |
| #2 | (SU %= '康复' + '康复训练' + '运动功能' + '步态分析' + '平衡训练' + '运动学分析' + '生理监测' + '康复疗效评估' + '姿势控制' + '震颤评估' + '运动迟缓评估') | 404,059 |
| #3 | (SU %= '帕金森病') | 38,166 |
| #4 | (SU %= '人工智能' + '机器学习' + '深度学习' + '神经网络' + '算法') | 2,760,662 |
| #5 | #1 AND #2 AND #3 AND #4 | 406 |

**WanFang**（7）

| **Line** | **Searches** | **NO.** |
| --- | --- | --- |
| #1 | (题名或关键词:(可穿戴电子设备) OR 题名或关键词:(外骨骼设备) OR 题名或关键词:(可穿戴) OR 题名或关键词:(可穿戴设备) OR 题名或关键词:(可穿戴传感器) OR 题名或关键词:(智能可穿戴) OR 题名或关键词:(智能服装) OR 题名或关键词:(智能衣物) OR 题名或关键词:(电子纺织品) OR 题名或关键词:(智能纺织品) OR 题名或关键词:(外骨骼) OR 题名或关键词:(可穿戴机器人) OR 题名或关键词:(可穿戴矫形器) OR 题名或关键词:(智能手表) OR 题名或关键词:(智能手环) OR 题名或关键词:(运动手环) OR 题名或关键词:(活动追踪器) OR 题名或关键词:(计步器) OR 题名或关键词:(智能鞋) OR 题名或关键词:(智能鞋垫)) | 28,745 |
| #2 | (题名或关键词:(康复) OR 题名或关键词:(康复训练) OR 题名或关键词:(运动功能) OR 题名或关键词:(运动功能评估) OR 题名或关键词:(运动功能测评) OR 题名或关键词:(步态分析) OR 题名或关键词:(平衡训练) OR 题名或关键词:(运动学分析) OR 题名或关键词:(生理监测) OR 题名或关键词:(康复疗效评估) OR 题名或关键词:(姿势控制) OR 题名或关键词:(震颤评估) OR 题名或关键词:(运动迟缓评估) OR 题名或关键词:(康复进展监测) OR 题名或关键词:(康复反馈)) | 615,483 |
| #3 | (题名或关键词:(帕金森病)) | 52,716 |
| #4 | (题名或关键词:(人工智能) OR 题名或关键词:(机器学习) OR 题名或关键词:(深度学习) OR 题名或关键词:(神经网络) OR 题名或关键词:(算法)) | 2,728,351 |
| #5 | #1 AND #2 AND #3 AND #4 | 8 |

**Sinomed**

| **Line** | **Searches** | **NO.** |
| --- | --- | --- |
| #1 | "可穿戴电子设备"[主题词] OR "可穿戴设备"[常用字段] OR "外骨骼设备"[常用字段] OR "可穿戴传感器"[常用字段] OR "智能可穿戴"[常用字段] OR "智能服装"[常用字段] OR "智能衣物"[常用字段] OR "电子纺织品"[常用字段] OR "智能纺织品"[常用字段] OR "外骨骼"[常用字段] OR "可穿戴机器人"[常用字段] OR "可穿戴矫形器"[常用字段] OR "智能手表"[常用字段] OR "运动手环"[常用字段] OR "活动追踪器"[常用字段] OR "计步器"[常用字段] OR "智能鞋"[常用字段] OR "智能鞋垫"[常用字段] | 2409 |
| #2 | "康复"[主题词] OR "康复训练"[常用字段] OR "运动功能"[常用字段] OR "运动功能评估"[常用字段] OR "运动功能测评"[常用字段] OR "步态分析"[常用字段] OR "平衡训练"[常用字段] OR "运动学分析"[常用字段] OR "生理监测"[常用字段] OR "康复疗效评估"[常用字段] OR "姿势控制"[常用字段] OR "震颤评估"[常用字段] OR "运动迟缓评估"[常用字段] OR "康复进展监测"[常用字段] OR "康复反馈"[常用字段] | 283,575 |
| #3 | "帕金森病"[主题词] OR "帕金森病"[常用字段] | 34,276 |
| #4 | "人工智能"[主题词] OR "人工智能"[常用字段] OR "机器学习"[常用字段] OR "深度学习"[常用字段] OR "神经网络"[常用字段] OR "算法"[常用字段] | 77,059 |
| #5 | #1 AND #2 AND #3 AND #4 | 181 |
