# Supplementary material for: AI-Enabled Wearables for Motor Function Assessment and Rehabilitation in Parkinson Disease: Scoping Review
Source: J Med Internet Res. 2026 Feb 26;28:e85596. doi: 10.2196/85596 (PMC12982951; doi:10.2196/85596)
Supplement: Multimedia Appendix 4 [file jmir_v28i1e85596_app4.docx]

**Appendix 4. Extracted data form**

| **1. Study details** | |
| --- | --- |
| Author | The author of the study. |
| Year of publication | The year in which the study was published. |
| Publication type | The publication medium. |
| Country/Region of publication | The country where the study was published. |
| Sample size | The number of participants from which the data was collected. |
| Age | The mean/range age of the participants |
| Sex distribution | The sex composition of participants. |
| Inclusion | The key inclusion criteria for participant enrollment. |
| Application Objective | The intended application of the study (e.g., monitoring/assessment, screening/state recognition, prediction, rehabilitation/feedback). |
| **2. Wearable device details** | |
| Type of WD | The device form factor/type. |
| Status of WD | The device status (commercial, prototype, or custom; include regulatory approvals if any). |
| Company of WD | The manufacturer or brand of the wearable device |
| Placement | The body location(s) where the device is worn. |
| OS | The operating system(s) used by the device and/or host. |
| Gateway | The intermediate device used to relay data. |
| Host | The computing environment where data are stored or processed. |
| Mode of data transfer | The data transmission mode. |
| Sensors in the wearables | The on-board sensors integrated into the device. |
| biosignalsa | The biosignals captured by the device. |
| Sensing type | The biosignal acquisition mode (passive or active). |
| Scenario | The usage scenario. |
| Duration of Monitoring/Intervention | The duration and frequency of monitoring or intervention. |
| **3. AI details** | |
| AI category | The broad AI category. |
| Task type | The computational task addressed. |
| algorithm | The specific algorithm(s) used. |
| Aim of AI algorithm | The application aim served by the algorithm (e.g., monitoring, screening, prediction, rehabilitation). |
| Validation approach | The model validation method. |
| Performance measures | The metrics used to evaluate performance. |
