# Supplementary material for: AI-Enabled Wearables for Motor Function Assessment and Rehabilitation in Parkinson Disease: Scoping Review
Source: J Med Internet Res. 2026 Feb 26;28:e85596. doi: 10.2196/85596 (PMC12982951; doi:10.2196/85596)
Supplement: Multimedia Appendix 5 [file jmir_v28i1e85596_app5.docx]

**Appendix 5. Quality assessment of the included studies.**

| Author and year | Screening | | Qualitative studies quality criteria | | | | | Randomized controlled trials quality criteria | | | | | Non-randomized studies quality criteria | | | | | Quantitative descriptive studies quality criteria | | | | | Mixed methods studies quality criteria | | | | | Score |
| --- | --- | --- | --- | --- | --- | --- | --- | --- | --- | --- | --- | --- | --- | --- | --- | --- | --- | --- | --- | --- | --- | --- | --- | --- | --- | --- | --- | --- |
|  | S1 | S2 | 1.1 | 1.2 | 1.3 | 1.4 | 1.5 | 2.1 | 2.2 | 2.3 | 2.4 | 2.5 | 3.1 | 3.2 | 3.3 | 3.4 | 3.5 | 4.1 | 4.2 | 4.3 | 4.4 | 4.5 | 5.1 | 5.2 | 5.3 | 5.4 | 5.5 |  |
| Aghanavesi et al. 2020 [1] | √ | √ |  |  |  |  |  |  |  |  |  |  | × | √ | × | × | √ |  |  |  |  |  |  |  |  |  |  | 40 |
| Li et al. 2022 [2] | √ | √ |  |  |  |  |  |  |  |  |  |  | × | √ | √ | × | √ |  |  |  |  |  |  |  |  |  |  | 60 |
| Ghayvat et al. 2025 [3] | √ | √ |  |  |  |  |  |  |  |  |  |  | × | √ | √ | × | √ |  |  |  |  |  |  |  |  |  |  | 60 |
| Delgado-Terán et al. 2025 [4] | √ | √ |  |  |  |  |  |  |  |  |  |  |  |  |  |  |  | √ | × | √ | × | √ |  |  |  |  |  | 60 |
| Liu et al. 2025 [5] | √ | √ |  |  |  |  |  |  |  |  |  |  | × | √ | √ | × | √ |  |  |  |  |  |  |  |  |  |  | 60 |
| Han et al. 2023 [6] | √ | √ |  |  |  |  |  |  |  |  |  |  | × | √ | √ | × | √ |  |  |  |  |  |  |  |  |  |  | 60 |
| Peraza et al. 2021 [7] | √ | √ |  |  |  |  |  |  |  |  |  |  |  |  |  |  |  | √ | × | √ | × | √ |  |  |  |  |  | 60 |
| Sanchez-Fernández et al. 2025 [8] | √ | √ |  |  |  |  |  |  |  |  |  |  |  |  |  |  |  | √ | × | √ | × | √ |  |  |  |  |  | 60 |
| Channa et al. 2022 [9] | √ | √ |  |  |  |  |  |  |  |  |  |  |  |  |  |  |  | √ | × | √ | × | √ |  |  |  |  |  | 60 |
| Sigcha et al. 2020 [10] | √ | √ |  |  |  |  |  |  |  |  |  |  |  |  |  |  |  | √ | × | √ | × | √ |  |  |  |  |  | 60 |
| Dvorani et al. 2024 [11] | √ | √ |  |  |  |  |  |  |  |  |  |  |  |  |  |  |  | × | √ | √ | × | √ |  |  |  |  |  | 60 |
| Bikias et al. 2021 [12] | √ | √ |  |  |  |  |  |  |  |  |  |  |  |  |  |  |  | √ | × | √ | × | √ |  |  |  |  |  | 60 |
| Aich et al. 2020 [13] | √ | √ |  |  |  |  |  |  |  |  |  |  | × | √ | √ | × | √ |  |  |  |  |  |  |  |  |  |  | 60 |
| Shi et al. 2022 [14] | √ | √ |  |  |  |  |  |  |  |  |  |  |  |  |  |  |  | √ | × | √ | √ | √ |  |  |  |  |  | 80 |
| Ezhilarasi et al. 2025 [15] |  |  |  |  |  |  |  |  |  |  |  |  |  |  |  |  |  | √ | × | √ | × | √ |  |  |  |  |  | 60 |
| Mahadevan et al. 2020 [16] | √ | √ |  |  |  |  |  |  |  |  |  |  | × | √ | √ | × | √ |  |  |  |  |  |  |  |  |  |  | 60 |
| Chan et al. 2024 [17] | √ | √ |  |  |  |  |  |  |  |  |  |  |  |  |  |  |  | √ | √ | √ | √ | √ |  |  |  |  |  | 100 |
| Pardoel et al. 2021 [18] | √ | √ |  |  |  |  |  |  |  |  |  |  |  |  |  |  |  | √ | × | √ | × | √ |  |  |  |  |  | 60 |
| Hssayeni et al. 2021 [19] | √ | √ |  |  |  |  |  |  |  |  |  |  |  |  |  |  |  | √ | × | √ | √ | √ |  |  |  |  |  | 80 |
| Tsakanikas et al. 2023 [20] | √ | √ |  |  |  |  |  |  |  |  |  |  |  |  |  |  |  | √ | × | √ | √ | √ |  |  |  |  |  | 80 |
| Brand et al. 2022 [21] | √ | √ |  |  |  |  |  |  |  |  |  |  |  |  |  |  |  | √ | × | √ | √ | √ |  |  |  |  |  | 80 |
| Pardoel et al. 2021 [22] | √ | √ |  |  |  |  |  |  |  |  |  |  |  |  |  |  |  | √ | × | √ | × | √ |  |  |  |  |  | 60 |
| Li et al. 2023 [23] | √ | √ |  |  |  |  |  |  |  |  |  |  | × | √ | √ | × | √ |  |  |  |  |  |  |  |  |  |  | 60 |
| Aghanavesi et al. 2020 [24] | √ | √ |  |  |  |  |  |  |  |  |  |  | × | √ | × | × | √ |  |  |  |  |  |  |  |  |  |  | 40 |
| Atrsaei et al. 2021 [25] | √ | √ |  |  |  |  |  |  |  |  |  |  |  |  |  |  |  | √ | × | √ | × | √ |  |  |  |  |  | 60 |
| Shawen et al. 2020 [26] | √ | √ |  |  |  |  |  |  |  |  |  |  |  |  |  |  |  | × | × | √ | × | √ |  |  |  |  |  | 40 |
| Naghavi et al. 2022 [27] | √ | √ |  |  |  |  |  |  |  |  |  |  |  |  |  |  |  | × | × | √ | × | √ |  |  |  |  |  | 40 |
| Zampogna et al. 2024 [28] | √ | √ |  |  |  |  |  |  |  |  |  |  |  |  |  |  |  | × | √ | √ | √ | √ |  |  |  |  |  | 80 |
| Reches et al. 2020 [29] | √ | √ |  |  |  |  |  |  |  |  |  |  |  |  |  |  |  | × | √ | √ | × | √ |  |  |  |  |  | 60 |
| Slemenšek et al. 2024 [30] | √ | √ |  |  |  |  |  |  |  |  |  |  | × | √ | √ | × | √ |  |  |  |  |  |  |  |  |  |  | 60 |
| Borzì et al. 2022 [31] | √ | √ |  |  |  |  |  |  |  |  |  |  |  |  |  |  |  | × | √ | √ | × | √ |  |  |  |  |  | 60 |
| Wang et al. 2024 [32] | √ | √ |  |  |  |  |  |  |  |  |  |  |  |  |  |  |  | × | × | √ | × | √ |  |  |  |  |  | 40 |
| Shuqair et al. 2024 [33] | √ | √ |  |  |  |  |  |  |  |  |  |  |  |  |  |  |  | × | √ | √ | × | √ |  |  |  |  |  | 60 |
| Kanellos et al. 2023 [34] | √ | √ |  |  |  |  |  |  |  |  |  |  |  |  |  |  |  | × | √ | √ | × | √ |  |  |  |  |  | 60 |
| Roth et al. 2021 [35] | √ | √ |  |  |  |  |  |  |  |  |  |  |  |  |  |  |  | × | √ | √ | × | √ |  |  |  |  |  | 60 |
| Pérez-Ibarra et al. 2020 [36] | √ | √ |  |  |  |  |  |  |  |  |  |  |  |  |  |  |  | × | √ | √ | × | √ |  |  |  |  |  | 60 |
| Meng et al. 2023 [37] | √ | √ |  |  |  |  |  |  |  |  |  |  |  |  |  |  |  | × | √ | √ | × | √ |  |  |  |  |  | 60 |
| Dattola et al. 2025 [38] | √ | √ |  |  |  |  |  |  |  |  |  |  |  |  |  |  |  | × | √ | √ | × | √ |  |  |  |  |  | 60 |
| Zhao et al. 2023 [39] | √ | √ |  |  |  |  |  |  |  |  |  |  |  |  |  |  |  | × | √ | √ | × | √ |  |  |  |  |  | 60 |
| Li et al. 2023 [40] | √ | √ |  |  |  |  |  |  |  |  |  |  |  |  |  |  |  | × | √ | √ | × | √ |  |  |  |  |  | 60 |
| Yan et al. 2024 [41] | √ | √ |  |  |  |  |  |  |  |  |  |  |  |  |  |  |  | × | × | √ | × | √ |  |  |  |  |  | 40 |
| Evers et al. 2025 [42] | √ | √ |  |  |  |  |  |  |  |  |  |  |  |  |  |  |  | √ | × | √ | × | √ |  |  |  |  |  | 60 |
| Greene et al. 2022 [43] | √ | √ |  |  |  |  |  |  |  |  |  |  |  |  |  |  |  | √ | × | √ | × | √ |  |  |  |  |  | 60 |
| Ricci et al. 2022 [44] | √ | √ |  |  |  |  |  |  |  |  |  |  | √ | √ | √ | × | √ |  |  |  |  |  |  |  |  |  |  | 80 |
| Ghosh et al. 2021 [45] | √ | √ |  |  |  |  |  |  |  |  |  |  |  |  |  |  |  | √ | × | √ | × | √ |  |  |  |  |  | 60 |
| Lin 2022 [46] | √ | √ |  |  |  |  |  |  |  |  |  |  |  |  |  |  |  | √ | × | √ | × | √ |  |  |  |  |  | 60 |
| Sun et al. 2022 [47] | √ | √ |  |  |  |  |  |  |  |  |  |  |  |  |  |  |  | × | × | √ | × | √ |  |  |  |  |  | 40 |
| Li et al. 2023 [48] | √ | √ |  |  |  |  |  |  |  |  |  |  |  |  |  |  |  | √ | × | √ | × | √ |  |  |  |  |  | 60 |
| Donisi et al. 2021 [49] | √ | √ |  |  |  |  |  |  |  |  |  |  | × | √ | √ | × | × |  |  |  |  |  |  |  |  |  |  | 40 |
| Sotirakis et al. 2024 [50] | √ | √ |  |  |  |  |  |  |  |  |  |  |  |  |  |  |  | √ | × | √ | √ | √ |  |  |  |  |  | 80 |
| Hong et al. 2025 [51] | √ | √ |  |  |  |  |  |  |  |  |  |  |  |  |  |  |  | √ | × | √ | √ | √ |  |  |  |  |  | 80 |
| Chen et al. 2023 [52] | √ | √ |  |  |  |  |  |  |  |  |  |  |  |  |  |  |  | √ | × | √ | × | √ |  |  |  |  |  | 60 |
| Channa et al. 2024 [53] | √ | √ |  |  |  |  |  |  |  |  |  |  |  |  |  |  |  | √ | × | √ | √ | √ |  |  |  |  |  | 80 |
| Singh et al. 2023 [54] | √ | √ |  |  |  |  |  |  |  |  |  |  |  |  |  |  |  | √ | √ | √ | × | √ |  |  |  |  |  | 80 |
| Weikert et al. 2025 [55] | √ | √ |  |  |  |  |  |  |  |  |  |  |  |  |  |  |  | √ | × | √ | × | √ |  |  |  |  |  | 60 |
| Yang et al. 2024 [56] | √ | √ |  |  |  |  |  |  |  |  |  |  |  |  |  |  |  | √ | × | √ | × | √ |  |  |  |  |  | 60 |
| Haddadi Esfahani et al. 2021 [57] | √ | √ |  |  |  |  |  |  |  |  |  |  |  |  |  |  |  | √ | × | √ | × | √ |  |  |  |  |  | 60 |
| Sun et al. 2024 [58] | √ | √ |  |  |  |  |  |  |  |  |  |  |  |  |  |  |  | √ | × | √ | × | √ |  |  |  |  |  | 60 |
| Dvorani et al. 2021 [59] | √ | √ |  |  |  |  |  |  |  |  |  |  |  |  |  |  |  | √ | × | √ | × | √ |  |  |  |  |  | 60 |
| Khan et al. 2025 [60] | √ | √ |  |  |  |  |  |  |  |  |  |  |  |  |  |  |  | √ | √ | √ | × | √ |  |  |  |  |  | 80 |
| Park et al. 2025 [61] | √ | √ |  |  |  |  |  |  |  |  |  |  |  |  |  |  |  | √ | √ | √ | × | √ |  |  |  |  |  | 80 |
| Goubault et al. 2025 [62] | √ | √ |  |  |  |  |  |  |  |  |  |  |  |  |  |  |  | √ | × | √ | × | √ |  |  |  |  |  | 60 |
| Liu et al. 2025 [63] | √ | √ |  |  |  |  |  |  |  |  |  |  |  |  |  |  |  | × | × | √ | × | √ |  |  |  |  |  | 40 |
| Nanayakkara et al. 2025 [64] | √ | √ |  |  |  |  |  |  |  |  |  |  |  |  |  |  |  | √ | √ | √ | × | √ |  |  |  |  |  | 80 |
| Ma et al. 2025 [65] | √ | √ |  |  |  |  |  |  |  |  |  |  |  |  |  |  |  | √ | √ | √ | × | √ |  |  |  |  |  | 80 |
| Lopes et al. 2025 [66] | √ | √ |  |  |  |  |  |  |  |  |  |  |  |  |  |  |  | √ | × | √ | × | √ |  |  |  |  |  | 60 |

**References**

1. Aghanavesi S, Westin J, Bergquist F, et al. A Multiple Motion Sensors Index for Motor State Quantification in Parkinson's Disease. Comput Methods Programs Biomed. 2020;189:105309. doi:10.1016/j.cmpb.2019.105309
2. Li Y, Bai Q, Yang X, et al. An Abnormal Gait Monitoring System for Patients With Parkinson's Disease Based on Wearable Devices. In: 2022 15th International Congress on Image and Signal Processing, BioMedical Engineering and Informatics (CISP-BMEI). Piscataway, NJ: IEEE; 2022:1-6. doi:10.1109/CISP-BMEI56279.2022.9980005
3. Ghayvat H, Awais M, Geddam R, et al. AiCareGaitRehabilitation: Multi-Modalities Sensor Data Fusion for AI-IoT–Enabled Real-Time Electrical Stimulation Device for Pre-FOG and Post-FOG to Person With Parkinson’s Disease. Inf Fusion. 2025;122:103155. doi:10.1016/j.inffus.2025.103155
4. Delgado-Terán JD, Hilbrants K, Mahmutović D, Silva de Lima AL, Wezel RJAV, Heida T. Ankle Sensor-Based Detection of Freezing of Gait in Parkinson's Disease in Semi-Free Living Environments. Sensors (Basel). 2025;25(6):1895. doi:10.3390/s25061895
5. Liu X, Zhang X, Li J, et al. Automated UPDRS Gait Scoring Using Wearable Sensor Fusion and Deep Learning. Bioengineering (Basel). 2025;12(7):686. doi:10.3390/bioengineering12070686
6. Han Y, Liu X, Zhang N, et al. Automatic Assessments of Parkinsonian Gait With Wearable Sensors for Human Assistive Systems. Sensors (Basel). 2023;23(4):2104. doi:10.3390/s23042104
7. Peraza LR, Kinnunen KM, McNaney R, et al. An Automatic Gait Analysis Pipeline for Wearable Sensors: A Pilot Study in Parkinson's Disease. Sensors (Basel). 2021;21(24):8286. doi:10.3390/s21248286
8. Sánchez-Fernández LP, Sánchez-Pérez LA, Martínez-Hernández JM. Computer Model for Gait Assessments in Parkinson's Patients Using a Fuzzy Inference Model and Inertial Sensors. Artif Intell Med. 2025;160:103059. doi:10.1016/j.artmed.2024.103059
9. Channa A, Popescu N, Faisal M. Parkinson's Disease Gait Evaluation Leveraging Wearable Insoles and a Deep Learning Approach. In: Proceedings of the 2022 8th International Conference on Control, Decision and Information Technologies (CoDIT). Piscataway, NJ: IEEE; 2022:543-549. doi:10.1109/CoDIT55151.2022.9804064
10. Sigcha L, Costa N, Pavón I, et al. Deep Learning Approaches for Detecting Freezing of Gait in Parkinson's Disease Patients Through On-Body Acceleration Sensors. Sensors (Basel). 2020;20(7):1895. doi:10.3390/s20071895
11. Dvorani A, Wiesener C, Salchow-Hommen C, et al. On-Demand Gait-Synchronous Electrical Cueing in Parkinson's Disease Using Machine Learning and Edge Computing: A Pilot Study. IEEE Open J Eng Med Biol. 2024;5:306-315. doi:10.1109/OJEMB.2024.3390562
12. Bikias T, Iakovakis D, Hadjidimitriou S, Charisis V, Hadjileontiadis LJ. DeepFoG: An IMU-Based Detection of Freezing of Gait Episodes in Parkinson's Disease Patients via Deep Learning. Front Robot AI. 2021;8:537384. doi:10.3389/frobt.2021.537384
13. Aich S, Pradhan PM, Chakraborty S, et al. Design of a Machine Learning-Assisted Wearable Accelerometer-Based Automated System for Studying the Effect of Dopaminergic Medicine on Gait Characteristics of Parkinson's Patients. J Healthc Eng. 2020;2020:1823268. doi:10.1155/2020/1823268
14. Shi B, Tay A, Au WL, Tan DML, Chia NSY, Yen SC. Detection of Freezing of Gait Using Convolutional Neural Networks and Data From Lower Limb Motion Sensors. IEEE Trans Biomed Eng. 2022;69(7):2256-2267. doi:10.1109/TBME.2022.3140258
15. Ezhilarasi J, Senthil Kumar T. Develop a novel, faster mask region-based convolutional neural network model with leave-one-subject-out to predict freezing of gait abnormalities of Parkinson’s disease. Neural Comput Appl. 2025;37(7):5441-57. doi:10.1007/s00521-024-10832-9.
16. Mahadevan N, Demanuele C, Zhang H, et al. Development of digital biomarkers for resting tremor and bradykinesia using a wrist-worn wearable device. NPJ Digit Med. 2020;3:5. Published 2020 Jan 15. doi:10.1038/s41746-019-0217-7
17. Chan LLY, Yang S, Aswani M, et al. Development, Validation, and Limits of Freezing of Gait Detection Using a Single Waist-Worn Device. IEEE Trans Biomed Eng. 2024;71(10):3024-3031. doi:10.1109/TBME.2024.3407059
18. Pardoel S, Shalin G, Nantel J, Lemaire ED, Kofman J. Early Detection of Freezing of Gait During Walking Using Inertial Measurement Unit and Plantar Pressure Distribution Data. Sensors (Basel). 2021;21(6):2246. doi:10.3390/s21062246
19. Hssayeni MD, Jimenez-Shahed J, Burack MA, Ghoraani B. Ensemble Deep Model for Continuous Estimation of Unified Parkinson's Disease Rating Scale III. Biomed Eng Online. 2021;20(1):32. doi:10.1186/s12938-021-00872-w
20. Tsakanikas V, Ntanis A, Rigas G, et al. Evaluating Gait Impairment in Parkinson's Disease From Instrumented Insole and IMU Sensor Data. Sensors (Basel). 2023;23(8):3902. doi:10.3390/s23083902
21. Brand YE, Schwartz D, Gazit E, Buchman AS, Gilad-Bachrach R, Hausdorff JM. Gait Detection From a Wrist-Worn Sensor Using Machine Learning Methods: A Daily Living Study in Older Adults and People With Parkinson's Disease. Sensors (Basel). 2022;22(18):7094. doi:10.3390/s22187094
22. Pardoel S, Shalin G, Lemaire ED, Kofman J, Nantel J. Grouping Successive Freezing of Gait Episodes Has Neutral to Detrimental Effect on Freeze Detection and Prediction in Parkinson's Disease. PLoS One. 2021;16(10):e0258544. doi:10.1371/journal.pone.0258544
23. Li D, Hallack A, Gwilym S, Li D, Hu MT, Cantley J. Investigating Gait-Responsive Somatosensory Cueing From a Wearable Device to Improve Walking in Parkinson's Disease. Biomed Eng Online. 2023;22(1):108. doi:10.1186/s12938-023-01167-y
24. Aghanavesi S, Bergquist F, Nyholm D, Senek M, Memedi M. Motion Sensor-Based Assessment of Parkinson's Disease Motor Symptoms During Leg Agility Tests: Results From Levodopa Challenge. IEEE J Biomed Health Inform. 2020;24(1):111-119. doi:10.1109/JBHI.2019.2898332
25. Atrsaei A, Hansen C, Elshehabi M, et al. Effect of Fear of Falling on Mobility Measured During Lab and Daily Activity Assessments in Parkinson's Disease. Front Aging Neurosci. 2021;13:722830. doi:10.3389/fnagi.2021.722830
26. Shawen N, O'Brien MK, Venkatesan S, et al. Role of Data Measurement Characteristics in the Accurate Detection of Parkinson's Disease Symptoms Using Wearable Sensors. J Neuroeng Rehabil. 2020;17(1):52. doi:10.1186/s12984-020-00684-4
27. Naghavi N, Wade E. Towards Real-Time Prediction of Freezing of Gait in Patients With Parkinson's Disease: A Novel Deep One-Class Classifier. IEEE J Biomed Health Inform. 2022;26(4):1726-1736. doi:10.1109/JBHI.2021.3103071
28. Zampogna A, Borzì L, Rinaldi D, et al. Unveiling the Unpredictable in Parkinson's Disease: Sensor-Based Monitoring of Dyskinesias and Freezing of Gait in Daily Life. Bioengineering (Basel). 2024;11(5):440. doi:10.3390/bioengineering11050440
29. Reches T, Dagan M, Herman T, et al. Using Wearable Sensors and Machine Learning to Automatically Detect Freezing of Gait During a FOG-Provoking Test. Sensors (Basel). 2020;20(16):4474. doi:10.3390/s20164474
30. Slemenšek J, Geršak J, Bratina B, van Midden VM, Pirtošek Z, Šafarič R. Wearable Online Freezing of Gait Detection and Cueing System. Bioengineering (Basel). 2024;11(10):1048. doi:10.3390/bioengineering11101048
31. Borzì L, Mazzetta I, Zampogna A, Suppa A, Irrera F, Olmo G. Predicting Axial Impairment in Parkinson's Disease Through a Single Inertial Sensor. Sensors (Basel). 2022;22(2):412. doi:10.3390/s22020412
32. Wang W, Lin J, Le X, et al. Addressing Multiple Challenges in Early Gait Freezing Prediction for Parkinson's Disease: A Practical Deep Learning Approach. IEEE J Biomed Health Inform. 2025;29(9):6251-6262. doi:10.1109/JBHI.2024.3522664
33. Shuqair M, Jimenez-Shahed J, Ghoraani B. Advancing Parkinson's Disease Management Through Multi-Shared-Task Self-Supervised Signal Processing. In: Proceedings of the 2024 58th Asilomar Conference on Signals, Systems, and Computers (ACSSC). Piscataway, NJ: IEEE; 2024:957-961. doi:10.1109/IEEECONF60004.2024.10942713
34. Kanellos FS, Tsamis KI, Rigas G, et al. Clinical Evaluation in Parkinson's Disease: Is the Golden Standard Shiny Enough? Sensors (Basel). 2023;23(8):3807. doi:10.3390/s23083807
35. Roth N, Küderle A, Ullrich M, et al. Hidden Markov Model–Based Stride Segmentation on Unsupervised Free-Living Gait Data in Parkinson's Disease Patients. J Neuroeng Rehabil. 2021;18(1):93. doi:10.1186/s12984-021-00883-7
36. Perez-Ibarra JC, Siqueira AAG, Krebs HI. Identification of Gait Events in Healthy Subjects and With Parkinson's Disease Using Inertial Sensors: An Adaptive Unsupervised Learning Approach. IEEE Trans Neural Syst Rehabil Eng. 2020;28(12):2933-2943. doi:10.1109/TNSRE.2020.3039999
37. Meng L, Pang J, Yang Y, Chen L, Xu R, Ming D. Inertial-Based Gait Metrics During Turning Improve the Detection of Early-Stage Parkinson's Disease Patients. IEEE Trans Neural Syst Rehabil Eng. 2023;31:1472-1482. doi:10.1109/TNSRE.2023.3237903
38. Dattola S, Ielo A, Quartarone A, De Cola MC. Integrating Wearable Sensor Signal Processing With Unsupervised Learning Methods for Tremor Classification in Parkinson's Disease. Bioengineering (Basel). 2025;12(1):37. doi:10.3390/bioengineering12010037
39. Zhao Y, Liu Y, Lu W, et al. Intelligent IoT anklets for monitoring the assessment of Parkinson’s disease. IEEE Sens J. 2023;23(24):31523-36. doi:10.1109/JSEN.2023.3331277.
40. Li Y, Yin J, Liu S, et al. Learning Hand Kinematics for Parkinson's Disease Assessment Using a Multimodal Sensor Glove. Adv Sci (Weinh). 2023;10(20):e2206982. doi:10.1002/advs.202206982
41. Yan F, Gong J, Zhang Q, He H. Learning Motion Primitives for the Quantification and Diagnosis of Mobility Deficits. IEEE Trans Biomed Eng. 2024;71(12):3339-3349. doi:10.1109/TBME.2024.3404357
42. Evers LJW, Raykov YP, Heskes TM, Krijthe JH, Bloem BR, Little MA. Passive Monitoring of Parkinson Tremor in Daily Life: A Prototypical Network Approach. Sensors (Basel). 2025;25(2):366. doi:10.3390/s25020366
43. Greene BR, Premoli I, McManus K, McGrath D, Caulfield B. Predicting Fall Counts Using Wearable Sensors: A Novel Digital Biomarker for Parkinson's Disease. Sensors (Basel). 2021;22(1):54. doi:10.3390/s22010054
44. Ricci M, Lazzaro GD, Errico V, Pisani A, Giannini F, Saggio G. The Impact of Wearable Electronics in Assessing the Effectiveness of Levodopa Treatment in Parkinson's Disease. IEEE J Biomed Health Inform. 2022;26(7):2920-2928. doi:10.1109/JBHI.2022.3160103
45. Ghosh N, Banerjee I. IoT-based freezing of gait detection using grey relational analysis. Internet Things. 2021;13:100068. doi:10.1016/j.iot.2019.100068.
46. Lin ZR. Quantitative assessment of Parkinson's motor symptoms based on machine learning. Sci Technol Innov Appl. 2022;12(34):50-4. doi:10.19981/j.CN23-1581/G3.2022.34.013.
47. Sun Y, Rong Z, Wang F, et al. Automatic quantitative study of motor function in patients with Parkinson's disease based on wearable inertial sensors. China Med Equip. 2023;38(10):27-32.
48. Li YM. Quantitative Assessment of Parkinson's Motor Symptoms Based on Deep Learning [dissertation]. Lanzhou, China: Lanzhou Jiaotong University; 2023. doi:10.27205/d.cnki.gltec.2023.000237
49. Donisi L, Cesarelli G, Balbi P, et al. Positive impact of short-term gait rehabilitation in Parkinson patients: a combined approach based on statistics and machine learning. Math Biosci Eng. 2021;18(5):6995-7009. doi:10.3934/mbe.2021348
50. Sotirakis C, Brzezicki MA, Patel S, Conway N, FitzGerald JJ, Antoniades CA. Predicting Future Fallers in Parkinson's Disease Using Kinematic Data Over a Period of 5 Years. NPJ Digit Med. 2024;7(1):345. doi:10.1038/s41746-024-01311-5
51. Hong G, Mao F, Zhang M, et al. Modeling and validation of wearable sensor-based gait parameters in Parkinson's disease patients with cognitive impairment. Front Aging Neurosci. 2025;17:1590224. doi:10.3389/fnagi.2025.1590224
52. Chen M, Sun Z, Xin T, Chen Y, Su F. An Interpretable Deep Learning Optimized Wearable Daily Detection System for Parkinson's Disease. IEEE Trans Neural Syst Rehabil Eng. 2023;31:3937-3946. doi:10.1109/TNSRE.2023.3314100
53. Channa A, Ruggeri G, Ifrim RC, et al. Cloud-Connected Bracelet for Continuous Monitoring of Parkinson’s Disease Patients: Integrating Advanced Wearable Technologies and Machine Learning. Electronics. 2024;13(6):1002. doi:10.3390/electronics13061002
54. Singh M, Prakash P, Kaur R, Sowers R, Brašić JR, Hernandez ME. A Deep Learning Approach for Automatic and Objective Grading of the Motor Impairment Severity in Parkinson's Disease for Use in Tele-Assessments. Sensors (Basel). 2023;23(21):9004. doi:10.3390/s23219004
55. Weikert T, Li Y, Paez-Granados D, Easthope CA. Automated Prediction of Item-Level ARAT Scores From Wearable Sensors. In: Proceedings of the 2025 IEEE International Conference on Rehabilitation Robotics (ICORR). Piscataway, NJ: IEEE; 2025:1239-1244. doi:10.1109/ICORR66766.2025.11063162
56. Yang PK, Filtjens B, Ginis P, et al. Freezing of gait assessment with inertial measurement units and deep learning: effect of tasks, medication states, and stops. J Neuroeng Rehabil. 2024;21(1):24. doi:10.1186/s12984-024-01320-1
57. Esfahani AH, Dyka Z, Ortmann S, Langendörfer P. Impact of Data Preparation in Freezing of Gait Detection Using Feature-Less Recurrent Neural Network. IEEE Access. 2021;9:138120-138131. doi:10.1109/ACCESS.2021.3117543
58. Sun H, Ye Q, Xia Y. Predicting Freezing of Gait in Patients With Parkinson’s Disease by Combination of Manually-Selected and Deep Learning Features. Biomed Signal Process Control. 2024;88:105639. doi:10.1016/j.bspc.2023.105639
59. Dvorani A, Waldheim V, Jochner MCE, et al. Real-Time Detection of Freezing Motions in Parkinson's Patients for Adaptive Gait Phase Synchronous Cueing. Front Neurol. 2021;12:720516. doi:10.3389/fneur.2021.720516
60. Khan U, Riaz Q, Hussain M, Zeeshan M, Krüger B. Towards Effective Parkinson’s Monitoring: Movement Disorder Detection and Symptom Identification Using Wearable Inertial Sensors. Algorithms. 2025;18(4):203. doi:10.3390/a18040203
61. Park H, Youm C, Cheon SM, et al. Using Machine Learning to Identify Parkinson's Disease Severity Subtypes With Multimodal Data. J Neuroeng Rehabil. 2025;22(1):126. doi:10.1186/s12984-025-01648-2
62. Goubault E, Martin C, Duval C, Daneault JF, Boissy P, Lebel K. Enhanced Detection and Segmentation of Sit Phases in Patients with Parkinson's Disease Using a Single SmartWatch and Random Forest Algorithms. Sensors (Basel). 2025;25(19):6104. Published 2025 Oct 3. doi:10.3390/s25196104
63. Liu T, Li Z, Ji X, et al. Friction nanogenerators based on asymmetrically adherent conductive hydrogels for multifunctional sensing and Parkinsonian gait diagnostics. Chemical Engineering Journal. 2025;169026.
64. Nanayakkara T, Herath HMKKMB, Malekroodi HS, Madusanka N, Yi M, Lee BI. Multi-Domain CoP Feature Analysis of Functional Mobility for Parkinson's Disease Detection Using Wearable Pressure Insoles. Sensors (Basel). 2025;25(18):5859. Published 2025 Sep 19. doi:10.3390/s25185859
65. Ma L, Lin S, Jin J, et al. Objective assessment of gait and posture symptoms in Parkinson's disease using wearable sensors and machine learning. Front Aging Neurosci. 2025;17:1618764. Published 2025 Aug 8. doi:10.3389/fnagi.2025.1618764
66. Lopes T, Reis Carneiro M, Morgadinho A, Reis Carneiro D, Tavakoli M. ParCuR-A Novel AI-Enabled Gait Cueing Wearable for Patients with Parkinson's Disease. Sensors (Basel). 2025;25(22):7077. Published 2025 Nov 20. doi:10.3390/s25227077
