# Supplementary material for: AI-Enabled Wearables for Motor Function Assessment and Rehabilitation in Parkinson Disease: Scoping Review
Source: J Med Internet Res. 2026 Feb 26;28:e85596. doi: 10.2196/85596 (PMC12982951; doi:10.2196/85596)
Supplement: Multimedia Appendix 8 [file jmir_v28i1e85596_app8.docx]

**Appendix 8. Detailed characteristics of AI algorithms used in the included wearable device studies for Parkinson’s disease.**

| **Study** | **AI category** | **Task type** | **AI algorithm** | **Aim of AI algorithm** | **Validation approach** | **Performance measures** |
| --- | --- | --- | --- | --- | --- | --- |
| Aghanavesi et al.[1] | ML | Regression | SVM | Monitoring or Assessment | k-fold CV | r; RMSE; ICC; Others (Bland–Altman) |
| Li et al.[2] | ML | Classification | RF; LR; GB | Monitoring or Assessment | k-fold CV | SEN; ACC; PREC; AUC; F1 |
| Ghayvat et al.[3] | ML and DL | Classification | SVM; CNN; MLP | Rehabilitation and Feedback | k-fold CV | SEN; ACC; PREC; AUC; F1 |
| Delgado-Terán et al.[4] | DL | Classification | CNN; MLP | State recognition or functional screening | LOOCV; k-fold CV | SEN; ACC; PREC; AUC; F1 |
| Liu et al.[5] | DL | Classification | CNN; LSTM; SA | Monitoring or Assessment | holdout | SEN; ACC; PREC |
| Han et al.[6] | ML | Regression; Classification | SVM; LR; NB; Others (LinReg) | Monitoring or Assessment | LOOCV | ACC; MAE |
| Peraza et al.[7] | DL | Regression; Classification | CNN; MLP | Monitoring or Assessment | LOOCV | r; MAE |
| Sánchez-Fernández et al.[8] | ML | Regression; Classification | SVM; RF; DT; Others (FIS) | Monitoring or Assessment | external validation | ACC |
| Channa et al.[9] | DL | Classification | CNN | Monitoring or Assessment | holdout | SEN; ACC; SPEC; PREC |
| Sigcha et al.[10] | ML and DL | Classification | SVM; CNN; RF; DT; LSTM; Others (DAE; OC-SVM) | Monitoring or Assessment | LOOCV; k-fold CV | SEN; SPEC; AUC; EER |
| Dvorani et al.[11] | ML | Classification | DT | Rehabilitation and Feedback | LOOCV | ACC |
| Bikias et al.[12] | DL | Classification | CNN; DT; XGB | State recognition or functional screening | LOOCV; k-fold CV | SEN; SPEC; F1 |
| Aich et al.[13] | ML | Classification | SVM; DT; KNN; NB | Monitoring or Assessment | k-fold CV; holdout | SEN; ACC; SPEC |
| Shi et al.[14] | DL | Classification | CNN | State recognition or functional screening | k-fold CV; holdout | SEN; ACC; PREC; F1; G-mean |
| Ezhilarasi et al.[15] | DL | Classification | CNN; LSTM | Prediction | LOOCV; k-fold CV | SEN; ACC; SPEC; AUC; F1; EER; G-mean; NPV; Others (Youden Index) |
| Mahadevan et al.[16] | ML | Regression; Classification | SVM; RF; LR | Monitoring or Assessment | LOOCV | SEN; ACC; SPEC; PREC; F1; r; RMSE |
| Chan et al.[17] | ML | Classification | CNN | State recognition or functional screening | k-fold CV | SEN; ACC; SPEC; PREC; F1; ICC; Others (Validation Loss) |
| Pardoel et al.[18] | ML | Classification | DT | Prediction | LOOCV | SEN; ACC; SPEC; F1; AUC |
| Hssayeni et al.[19] | DL | Regression | CNN; LSTM | Monitoring or Assessment | LOOCV | r; MAE |
| Tsakanikas et al.[20] | ML | Classification | SVM; RF; DT; GB | Monitoring or Assessment | k-fold CV | SEN; ACC; F1; AUC |
| Brand et al.[21] | ML | Classification | CNN; LSTM; HMM | Monitoring or Assessment | holdout; k-fold CV | SEN; ACC; PREC; AUC; r |
| Pardoel et al.[22] | ML | Classification | DT | Monitoring or Assessment | LOOCV | SEN; SPEC; PREC; Others (Identification Delay; Episodes Identified) |
| Li et al.[23] | ML | Classification | RF | Rehabilitation and Feedback | LOOCV | SEN; SPEC; ROC |
| Aghanavesi et al.[24] | ML | Regression | SVM; DT; LR | Monitoring or Assessment | k-fold CV | r; RMSE |
| Atrsaei et al.[25] | ML | Classification | DT; LR | State recognition or functional screening | LOOCV; external validation | SEN; ACC; SPEC; PREC; AUC |
| Shawen et al.[26] | ML | Classification | RF | Monitoring or Assessment | LOOCV; holdout | AUC; ROC |
| Naghavi et al.[27] | DL | Classification | Others (Deep one-class classification) | Prediction | LOOCV | SEN; SPEC; AUC |
| Zampogna et al.[28] | ML | Classification | SVM; LR | Monitoring or Assessment | LOOCV | SEN; ACC; SPEC; PREC; AUC; NPV |
| Reches et al.[29] | ML | Classification | SVM | State recognition or functional screening | LOOCV; holdout | SEN; SPEC; PREC; ROC; Others (Percent time frozen; Number of FOG episodes) |
| Slemenšek et al.[30] | DL | Classification | CNN; Others (RNN) | Rehabilitation and Feedback | LOOCV | SEN; ACC; SPEC; PREC; F1; Others (Detection delay) |
| Borzìet al.[31] | ML | Regression | SVM | Prediction | LOOCV | r; MAE; RMSE |
| Wang et al.[32] | DL | Classification | CNN; LSTM; SA | Prediction | k-fold CV | SEN; ACC; SPEC; AUC |
| Shuqair et al.[33] | DL | Regression | CNN; LSTM | Monitoring or Assessment | LOOCV | r; MAE; R² |
| Kanellos et al.[34] | ML | Regression; Classification | LR | Monitoring or Assessment | external validation | r |
| Roth et al.[35] | ML | Classification | HMM | Monitoring or Assessment | LOOCV | SEN |
| Perez-Ibarra et al.[36] | ML | Clustering; Regression; | HMM | Monitoring or Assessment | LOOCV | SEN; F1 |
| Meng et al.[37] | ML | Classification | SVM | State recognition or functional screening | k-fold CV | ACC; AUC; ROC |
| Dattola et al.[38] | ML | Classification | K-means | State recognition or functional screening | NR | SEN; ACC; PREC; F1 |
| Zhao et al.[39] | ML | Classification | SVM; KNN; DT; NB; AB | State recognition or functional screening | k-fold CV | SEN; ACC; SPEC; PREC; F1 |
| Li et al.[40] | ML | Clustering; Classification | MLP; K-means | State recognition or functional screening | k-fold CV | SEN; ACC; PREC; F1; ICC; Kappa |
| Yan et al.[41] | DL | Classification | SVM; CNN; LR; MLP; RBF; Others (Dictionary learning) | State recognition or functional screening | holdout | SEN; SPEC; PREC; AUC; F1 |
| Evers et al.[42] | DL | Classification | RF; LR; RBF; Others (ProtoNet) | Monitoring or Assessment | LOOCV | SEN; SPEC; AUC; r |
| Greene et al.[43] | ML | Regression | RF; LR; EN; Others (PoisReg; Ensemble Learning) | Prediction | k-fold CV; holdout | r; RMSE |
| Ricci et al.[44] | ML | Classification | SVM | Monitoring or Assessment | LOOCV | r |
| Ghosh et al.[45] | ML | Classification | SVM; RF; DT; Others (Bagging) | State recognition or functional screening | holdout | SEN; ACC; SPEC; AUC; F1; Others (MCC) |
| Lin et al.[46] | ML | Classification | SVM; RF; KNN | Monitoring or Assessment | LOOCV | SEN; ACC; SPEC |
| Sun et al.[47] | ML | Classification | SVM; DT | Monitoring or Assessment | k-fold CV | SEN; ACC; PREC; F1 |
| Li et al.[48] | ML and DL | Classification | SVM; CNN; LSTM; SA | Monitoring or Assessment | k-fold CV | SEN; ACC; PREC; F1 |
| Donisi et al.[49] | ML | Classification | RF; DT; GB; Others (Rotation Forest) | Rehabilitation and Feedback | LOOCV | ACC; AUC |
| Sotirakis et al.[50] | ML | Classification | SVM; RF; LR; XGB; EN | Prediction | k-fold CV | SEN; ACC; PREC; AUC; F1 |
| Hong et al.[51] | ML | Classification | SVM; DT; LR; MLP; KNN; NB | Monitoring or Assessment | k-fold CV | AUC; r; Others (DCA; Calibration Curve) |
| Chen et al.[52] | DL | Classification | CNN; LR | State recognition or functional screening | k-fold CV | ACC; ROC |
| Channa et al.[53] | DL | Classification | CNN | Monitoring or Assessment | holdout; k-fold CV | SEN; ACC |
| Singh et al.[54] | DL | Classification | CNN | Monitoring or Assessment | k-fold CV | ACC |
| Weikert et al.[55] | ML | Classification | SVM; RF; LR; MLP; KNN; AB | Monitoring or Assessment | k-fold CV | ACC; PREC; AUC; F1 |
| Yang et al.[56] | ML and DL | Classification | SVM; CNN; LSTM; KNN; XGB | Monitoring or Assessment | LOOCV | F1; ICC |
| Esfahani et al.[57] | DL | Classification | LSTM | State recognition or functional screening | holdout | SEN; SPEC; AUC; ROC |
| Sun et al.[58] | ML and DL | Classification | CNN; RF | Prediction | LOOCV | SEN; ACC; SPEC; PREC; F1; Kappa |
| Dvorani et al.[59] | ML | Classification | SVM; AB | Rehabilitation and Feedback | LOOCV | SEN; ACC; SPEC; AUC |
| Khan et al.[60] | ML and DL | Classification | CNN; RF; Others (GRU) | Monitoring or Assessment | NR | SEN; ACC; PREC |
| Park et al.[61] | ML | Clustering; Regression; Classification | RF; LR; K-means; EN | Monitoring or Assessment | NR | ACC; AUC; ROC; MAE; R² |
| Goubault et al. [62] | ML | Classification | RF | Monitoring or Assessment | k-fold CV | SEN; SPEC; F1; Others (Time difference ΔT) |
| Liu et al. [63] | DL | Classification | CNN; LSTM; MLP | State recognition or functional screening | holdout | ACC |
| Nanayakkara et al.[64] | ML | Classification | SVM; LR; RF; KNN; NB | State recognition or functional screening | k-fold CV; holdout | SEN; ACC; PREC; F1; AUC |
| Ma et al.[65] | ML | Regression; Classification | XGB; SVM; Others (LASSO) | Monitoring or Assessment | LOOCV; holdout | ACC; F1; Kappa; MAE; RMSE; r |
| Lopes et al. [66] | ML | Classification | SVM | State recognition or functional screening | LOOCV; k-fold CV | SEN; SPEC; F1 |

**ML=** Machine Learning; **DL=** Deep Learning; **SVM=** Support Vector Machine; **RF=** Random Forest; **LR=** Logistic Regression; **GB=** Gradient Boosting; **CNN=** Convolutional Neural Network; **MLP=** Multilayer Perceptron; **LSTM=** Long Short-Term Memory; **SA=** Self-Attention; **NB=** Naive Bayes; **LinReg=** Linear Regression; **DT=** Decision Tree; **FIS=** Fuzzy Inference System; **DAE=** Denoising Autoencoder; **OC-SVM=** One-Class Support Vector Machine; **XGB=** Extreme Gradient Boosting (XGBoost); **KNN=** k-Nearest Neighbors; **HMM=** Hidden Markov Model; **RNN=** Recurrent Neural Network; **K-means=** K-means Clustering; **AB=** AdaBoost; **RBF=** Radial Basis Function; **ProtoNet=** Prototypical Networks; **EN=** Elastic Net; **PoisReg=** Poisson Regression; **GRU=** Gated Recurrent Unit; **LOOCV**= Leave-One-Out Cross-Validation; **k-fold CV**= k-Fold Cross-Validation; **holdout**= Hold-out validation; **SEN**= Sensitivity; **ACC**= Accuracy; **SPEC**= Specificity; **PREC**= Precision; **AUC**= Area Under the Curve; **F1**= F1-score; **r**= Correlation Coefficient; **ROC**= Receiver Operating Characteristic; **MAE**= Mean Absolute Error; **RMSE**= Root Mean Square Error; **ICC**= Intraclass Correlation Coefficient; **EER**= Equal Error Rate; **NPV**= Negative Predictive Value; **R^2^**= Coefficient of Determination; **Kappa**= Cohen's Kappa; **MCC**= Matthews Correlation Coefficient; **DCA**= Decision Curve Analysis; **LASSO** = Least Absolute Shrinkage and Selection Operator; **NR**= Not Reported**References：**

1. Aghanavesi S, Westin J, Bergquist F, et al. A multiple motion sensors index for motor state quantification in Parkinson's disease. Comput Methods Programs Biomed. 2020;189:105309. doi:10.1016/j.cmpb.2019.105309
2. Li Y, Bai Q, Yang X, et al. An abnormal gait monitoring system for patients with Parkinson's disease based on wearable devices[C]// 2022 15th International Congress on Image and Signal Processing, BioMedical Engineering and Informatics (CISP-BMEI). IEEE; 2022:1-6. doi:10.1109/CISP-BMEI56279.2022.9980005.
3. Ghayvat H, Awais M, Geddam R, et al. AiCareGaitRehabilitation: Multi-modalities sensor data fusion for AI-IoT enabled real-time electrical stimulation device for pre-FOG and post-FOG to person with Parkinson’s disease. Inf Fusion. 2025;122:103155. doi:10.1016/j.inffus.2025.103155.
4. Delgado-Terán JD, Hilbrants K, Mahmutović D, Silva de Lima AL, Wezel RJAV, Heida T. Ankle Sensor-Based Detection of Freezing of Gait in Parkinson's Disease in Semi-Free Living Environments. Sensors (Basel). 2025;25(6):1895. Published 2025 Mar 18. doi:10.3390/s25061895、
5. Liu X, Zhang X, Li J, et al. Automated UPDRS Gait Scoring Using Wearable Sensor Fusion and Deep Learning. Bioengineering (Basel). 2025;12(7):686. Published 2025 Jun 24. doi:10.3390/bioengineering12070686
6. Han Y, Liu X, Zhang N, et al. Automatic Assessments of Parkinsonian Gait with Wearable Sensors for Human Assistive Systems. Sensors (Basel). 2023;23(4):2104. Published 2023 Feb 13. doi:10.3390/s23042104
7. Peraza LR, Kinnunen KM, McNaney R, et al. An Automatic Gait Analysis Pipeline for Wearable Sensors: A Pilot Study in Parkinson's Disease. Sensors (Basel). 2021;21(24):8286. Published 2021 Dec 11. doi:10.3390/s21248286
8. Sánchez-Fernández LP, Sánchez-Pérez LA, Martínez-Hernández JM. Computer model for gait assessments in Parkinson's patients using a fuzzy inference model and inertial sensors. Artif Intell Med. 2025;160:103059. doi:10.1016/j.artmed.2024.103059
9. Channa A, Popescu N, Faisal M. Parkinson's disease gait evaluation leveraging wearable insoles and deep learning approach[C]// 2022 8th International Conference on Control, Decision and Information Technologies (CoDIT). IEEE; 2022:543-9. doi: 10.1109/CoDIT55151.2022.9804064.
10. Sigcha L, Costa N, Pavón I, et al. Deep Learning Approaches for Detecting Freezing of Gait in Parkinson's Disease Patients through On-Body Acceleration Sensors. Sensors (Basel). 2020;20(7):1895. Published 2020 Mar 29. doi:10.3390/s20071895
11. Dvorani A, Wiesener C, Salchow-Hommen C, et al. On-Demand Gait-Synchronous Electrical Cueing in Parkinson's Disease Using Machine Learning and Edge Computing: A Pilot Study. IEEE Open J Eng Med Biol. 2024;5:306-315. Published 2024 Apr 18. doi:10.1109/OJEMB.2024.3390562
12. Bikias T, Iakovakis D, Hadjidimitriou S, Charisis V, Hadjileontiadis LJ. DeepFoG: An IMU-Based Detection of Freezing of Gait Episodes in Parkinson's Disease Patients via Deep Learning. Front Robot AI. 2021;8:537384. Published 2021 May 7. doi:10.3389/frobt.2021.537384
13. Aich S, Pradhan PM, Chakraborty S, et al. Design of a Machine Learning-Assisted Wearable Accelerometer-Based Automated System for Studying the Effect of Dopaminergic Medicine on Gait Characteristics of Parkinson's Patients. J Healthc Eng. 2020;2020:1823268. Published 2020 Feb 18. doi:10.1155/2020/1823268
14. Shi B, Tay A, Au WL, Tan DML, Chia NSY, Yen SC. Detection of Freezing of Gait Using Convolutional Neural Networks and Data From Lower Limb Motion Sensors. IEEE Trans Biomed Eng. 2022;69(7):2256-2267. doi:10.1109/TBME.2022.3140258
15. Ezhilarasi J, Senthil Kumar T. Develop a novel, faster mask region-based convolutional neural network model with leave-one-subject-out to predict freezing of gait abnormalities of Parkinson’s disease. Neural Comput Appl. 2025;37(7):5441-57. doi:10.1007/s00521-024-10832-9.
16. Mahadevan N, Demanuele C, Zhang H, et al. Development of digital biomarkers for resting tremor and bradykinesia using a wrist-worn wearable device. NPJ Digit Med. 2020;3:5. Published 2020 Jan 15. doi:10.1038/s41746-019-0217-7
17. Chan LLY, Yang S, Aswani M, et al. Development, Validation, and Limits of Freezing of Gait Detection Using a Single Waist-Worn Device. IEEE Trans Biomed Eng. 2024;71(10):3024-3031. doi:10.1109/TBME.2024.3407059
18. Pardoel S, Shalin G, Nantel J, Lemaire ED, Kofman J. Early Detection of Freezing of Gait during Walking Using Inertial Measurement Unit and Plantar Pressure Distribution Data. Sensors (Basel). 2021;21(6):2246. Published 2021 Mar 23. doi:10.3390/s21062246
19. Hssayeni MD, Jimenez-Shahed J, Burack MA, Ghoraani B. Ensemble deep model for continuous estimation of Unified Parkinson's Disease Rating Scale III. Biomed Eng Online. 2021;20(1):32. Published 2021 Mar 31. doi:10.1186/s12938-021-00872-w
20. Tsakanikas V, Ntanis A, Rigas G, et al. Evaluating Gait Impairment in Parkinson's Disease from Instrumented Insole and IMU Sensor Data. Sensors (Basel). 2023;23(8):3902. Published 2023 Apr 12. doi:10.3390/s23083902
21. Brand YE, Schwartz D, Gazit E, Buchman AS, Gilad-Bachrach R, Hausdorff JM. Gait Detection from a Wrist-Worn Sensor Using Machine Learning Methods: A Daily Living Study in Older Adults and People with Parkinson's Disease. Sensors (Basel). 2022;22(18):7094. Published 2022 Sep 19. doi:10.3390/s22187094
22. Pardoel S, Shalin G, Lemaire ED, Kofman J, Nantel J. Grouping successive freezing of gait episodes has neutral to detrimental effect on freeze detection and prediction in Parkinson's disease. PLoS One. 2021;16(10):e0258544. Published 2021 Oct 12. doi:10.1371/journal.pone.0258544
23. Li D, Hallack A, Gwilym S, Li D, Hu MT, Cantley J. Investigating gait-responsive somatosensory cueing from a wearable device to improve walking in Parkinson's disease. Biomed Eng Online. 2023;22(1):108. Published 2023 Nov 16. doi:10.1186/s12938-023-01167-y
24. Aghanavesi S, Bergquist F, Nyholm D, Senek M, Memedi M. Motion Sensor-Based Assessment of Parkinson's Disease Motor Symptoms During Leg Agility Tests: Results From Levodopa Challenge. IEEE J Biomed Health Inform. 2020;24(1):111-119. doi:10.1109/JBHI.2019.2898332
25. Atrsaei A, Hansen C, Elshehabi M, et al. Effect of Fear of Falling on Mobility Measured During Lab and Daily Activity Assessments in Parkinson's Disease. Front Aging Neurosci. 2021;13:722830. Published 2021 Nov 30. doi:10.3389/fnagi.2021.722830
26. Shawen N, O'Brien MK, Venkatesan S, et al. Role of data measurement characteristics in the accurate detection of Parkinson's disease symptoms using wearable sensors. J Neuroeng Rehabil. 2020;17(1):52. Published 2020 Apr 20. doi:10.1186/s12984-020-00684-4
27. Naghavi N, Wade E. Towards Real-Time Prediction of Freezing of Gait in Patients With Parkinson's Disease: A Novel Deep One-Class Classifier. IEEE J Biomed Health Inform. 2022;26(4):1726-1736. doi:10.1109/JBHI.2021.3103071
28. Zampogna A, Borzì L, Rinaldi D, et al. Unveiling the Unpredictable in Parkinson's Disease: Sensor-Based Monitoring of Dyskinesias and Freezing of Gait in Daily Life. Bioengineering (Basel). 2024;11(5):440. Published 2024 Apr 29. doi:10.3390/bioengineering11050440
29. Reches T, Dagan M, Herman T, et al. Using Wearable Sensors and Machine Learning to Automatically Detect Freezing of Gait during a FOG-Provoking Test. Sensors (Basel). 2020;20(16):4474. Published 2020 Aug 10. doi:10.3390/s20164474
30. Slemenšek J, Geršak J, Bratina B, van Midden VM, Pirtošek Z, Šafarič R. Wearable Online Freezing of Gait Detection and Cueing System. Bioengineering (Basel). 2024;11(10):1048. Published 2024 Oct 20. doi:10.3390/bioengineering11101048
31. Borzì L, Mazzetta I, Zampogna A, Suppa A, Irrera F, Olmo G. Predicting Axial Impairment in Parkinson's Disease through a Single Inertial Sensor. Sensors (Basel). 2022;22(2):412. Published 2022 Jan 6. doi:10.3390/s22020412
32. Wang W, Lin J, Le X, et al. Addressing Multiple Challenges in Early Gait Freezing Prediction for Parkinson's Disease: A Practical Deep Learning Approach. IEEE J Biomed Health Inform. 2025;29(9):6251-6262. doi:10.1109/JBHI.2024.3522664
33. Shuqair M, Jimenez-Shahed J, Ghoraani B. Advancing Parkinson's disease management through multi-shared-task self-supervised signal processing[C]// 2024 58th Asilomar Conference on Signals, Systems, and Computers. IEEE; 2024:957-61. doi:10.1109/IEEECONF60004.2024.10942713.
34. Kanellos FS, Tsamis KI, Rigas G, et al. Clinical Evaluation in Parkinson's Disease: Is the Golden Standard Shiny Enough?. Sensors (Basel). 2023;23(8):3807. Published 2023 Apr 7. doi:10.3390/s23083807
35. Roth N, Küderle A, Ullrich M, et al. Hidden Markov Model based stride segmentation on unsupervised free-living gait data in Parkinson's disease patients. J Neuroeng Rehabil. 2021;18(1):93. Published 2021 Jun 3. doi:10.1186/s12984-021-00883-7
36. Perez-Ibarra JC, Siqueira AAG, Krebs HI. Identification of Gait Events in Healthy Subjects and With Parkinson's Disease Using Inertial Sensors: An Adaptive Unsupervised Learning Approach. IEEE Trans Neural Syst Rehabil Eng. 2020;28(12):2933-2943. doi:10.1109/TNSRE.2020.3039999
37. Meng L, Pang J, Yang Y, Chen L, Xu R, Ming D. Inertial-Based Gait Metrics During Turning Improve the Detection of Early-Stage Parkinson's Disease Patients. IEEE Trans Neural Syst Rehabil Eng. 2023;31:1472-1482. doi:10.1109/TNSRE.2023.3237903
38. Dattola S, Ielo A, Quartarone A, De Cola MC. Integrating Wearable Sensor Signal Processing with Unsupervised Learning Methods for Tremor Classification in Parkinson's Disease. Bioengineering (Basel). 2025;12(1):37. Published 2025 Jan 6. doi:10.3390/bioengineering12010037
39. Zhao Y, Liu Y, Lu W, et al. Intelligent IoT anklets for monitoring the assessment of Parkinson’s disease. IEEE Sens J. 2023;23(24):31523-36. doi:10.1109/JSEN.2023.3331277.
40. Li Y, Yin J, Liu S, et al. Learning Hand Kinematics for Parkinson's Disease Assessment Using a Multimodal Sensor Glove. Adv Sci (Weinh). 2023;10(20):e2206982. doi:10.1002/advs.202206982
41. Yan F, Gong J, Zhang Q, He H. Learning Motion Primitives for the Quantification and Diagnosis of Mobility Deficits. IEEE Trans Biomed Eng. 2024;71(12):3339-3349. doi:10.1109/TBME.2024.3404357
42. Evers LJW, Raykov YP, Heskes TM, Krijthe JH, Bloem BR, Little MA. Passive Monitoring of Parkinson Tremor in Daily Life: A Prototypical Network Approach. Sensors (Basel). 2025;25(2):366. Published 2025 Jan 9. doi:10.3390/s25020366
43. Greene BR, Premoli I, McManus K, McGrath D, Caulfield B. Predicting Fall Counts Using Wearable Sensors: A Novel Digital Biomarker for Parkinson's Disease. Sensors (Basel). 2021;22(1):54. Published 2021 Dec 22. doi:10.3390/s22010054
44. Ricci M, Lazzaro GD, Errico V, Pisani A, Giannini F, Saggio G. The Impact of Wearable Electronics in Assessing the Effectiveness of Levodopa Treatment in Parkinson's Disease. IEEE J Biomed Health Inform. 2022;26(7):2920-2928. doi:10.1109/JBHI.2022.3160103
45. Ghosh N, Banerjee I. IoT-based freezing of gait detection using grey relational analysis. Internet Things. 2021;13:100068. doi:10.1016/j.iot.2019.100068.
46. Lin ZR. Quantitative assessment of Parkinson's motor symptoms based on machine learning. Sci Technol Innov Appl. 2022;12(34):50-4. doi:10.19981/j.CN23-1581/G3.2022.34.013.
47. Sun Y, Rong Z, Wang F, et al. Automatic quantitative study of motor function in patients with Parkinson's disease based on wearable inertial sensors. China Med Equip. 2023;38(10):27-32.
48. Li YM. Quantitative assessment of Parkinson's motor symptoms based on deep learning [dissertation]. Lanzhou, China: Lanzhou Jiaotong University; 2023. doi:10.27205/d.cnki.gltec.2023.000237.
49. Donisi L, Cesarelli G, Balbi P, et al. Positive impact of short-term gait rehabilitation in Parkinson patients: a combined approach based on statistics and machine learning. Math Biosci Eng. 2021;18(5):6995-7009. doi:10.3934/mbe.2021348
50. Sotirakis C, Brzezicki MA, Patel S, Conway N, FitzGerald JJ, Antoniades CA. Predicting future fallers in Parkinson's disease using kinematic data over a period of 5 years. NPJ Digit Med. 2024;7(1):345. Published 2024 Dec 5. doi:10.1038/s41746-024-01311-5
51. Hong G, Mao F, Zhang M, et al. Modeling and validation of wearable sensor-based gait parameters in Parkinson's disease patients with cognitive impairment. Front Aging Neurosci. 2025;17:1590224. Published 2025 Jul 25. doi:10.3389/fnagi.2025.1590224
52. Chen M, Sun Z, Xin T, Chen Y, Su F. An Interpretable Deep Learning Optimized Wearable Daily Detection System for Parkinson's Disease. IEEE Trans Neural Syst Rehabil Eng. 2023;31:3937-3946. doi:10.1109/TNSRE.2023.3314100
53. Channa A, Ruggeri G, Ifrim RC, et al. Cloud-connected bracelet for continuous monitoring of Parkinson’s disease patients: integrating advanced wearable technologies and machine learning. Electronics. 2024;13(6):1002. doi:10.3390/electronics13061002.
54. Singh M, Prakash P, Kaur R, Sowers R, Brašić JR, Hernandez ME. A Deep Learning Approach for Automatic and Objective Grading of the Motor Impairment Severity in Parkinson's Disease for Use in Tele-Assessments. Sensors (Basel). 2023;23(21):9004. Published 2023 Nov 6. doi:10.3390/s23219004
55. Weikert T, Li Y, Paez-Granados D, Easthope CA. Automated Prediction of Item-Level Arat Scores From Wearable Sensors. IEEE Int Conf Rehabil Robot. 2025;2025:1239-1244. doi:10.1109/ICORR66766.2025.11063162
56. Yang PK, Filtjens B, Ginis P, et al. Freezing of gait assessment with inertial measurement units and deep learning: effect of tasks, medication states, and stops. J Neuroeng Rehabil. 2024;21(1):24. Published 2024 Feb 13. doi:10.1186/s12984-024-01320-1
57. Esfahani AH, Dyka Z, Ortmann S, Langendörfer P. Impact of data preparation in freezing of gait detection using feature-less recurrent neural network. IEEE Access. 2021;9:138120-138131.
58. Sun H, Ye Q, Xia Y. Predicting freezing of gait in patients with Parkinson’s disease by combination of manually-selected and deep learning features. Biomed Signal Process Control. 2024;88:105639.
59. Dvorani A, Waldheim V, Jochner MCE, et al. Real-Time Detection of Freezing Motions in Parkinson's Patients for Adaptive Gait Phase Synchronous Cueing. Front Neurol. 2021;12:720516. Published 2021 Dec 6. doi:10.3389/fneur.2021.720516
60. Khan U, Riaz Q, Hussain M, et al. Towards effective Parkinson’s monitoring: movement disorder detection and symptom identification using wearable inertial sensors. Algorithms. 2025;18(4):192.
61. Park H, Youm C, Cheon SM, et al. Using machine learning to identify Parkinson's disease severity subtypes with multimodal data. J Neuroeng Rehabil. 2025;22(1):126. Published 2025 Jun 2. doi:10.1186/s12984-025-01648
62. Goubault E, Martin C, Duval C, Daneault JF, Boissy P, Lebel K. Enhanced Detection and Segmentation of Sit Phases in Patients with Parkinson's Disease Using a Single SmartWatch and Random Forest Algorithms. Sensors (Basel). 2025;25(19):6104. Published 2025 Oct 3. doi:10.3390/s25196104
63. Liu T, Li Z, Ji X, et al. Friction nanogenerators based on asymmetrically adherent conductive hydrogels for multifunctional sensing and Parkinsonian gait diagnostics. Chemical Engineering Journal. 2025;169026.
64. Nanayakkara T, Herath HMKKMB, Malekroodi HS, Madusanka N, Yi M, Lee BI. Multi-Domain CoP Feature Analysis of Functional Mobility for Parkinson's Disease Detection Using Wearable Pressure Insoles. Sensors (Basel). 2025;25(18):5859. Published 2025 Sep 19. doi:10.3390/s25185859
65. Ma L, Lin S, Jin J, et al. Objective assessment of gait and posture symptoms in Parkinson's disease using wearable sensors and machine learning. Front Aging Neurosci. 2025;17:1618764. Published 2025 Aug 8. doi:10.3389/fnagi.2025.1618764
66. Lopes T, Reis Carneiro M, Morgadinho A, Reis Carneiro D, Tavakoli M. ParCuR-A Novel AI-Enabled Gait Cueing Wearable for Patients with Parkinson's Disease. Sensors (Basel). 2025;25(22):7077. Published 2025 Nov 20. doi:10.3390/s25227077
