# Supplementary material for: AI-Enabled Wearables for Motor Function Assessment and Rehabilitation in Parkinson Disease: Scoping Review
Source: J Med Internet Res. 2026 Feb 26;28:e85596. doi: 10.2196/85596 (PMC12982951; doi:10.2196/85596)
Supplement: Multimedia Appendix 9 [file jmir_v28i1e85596_app9.docx]

**Appendix 9. Challenges and opportunities of AI-enabled wearable devices in Parkinson’s disease research.**

| **Challenges** | **Opportunities** |
| --- | --- |
| **Evidence and data quality** | |
| - Short follow-up and single-session/short-term monitoring; limited longitudinal real-world data. - Single-centre convenience samples; limited representativeness (sex/age strata, special subgroups). - External validation is scarce; calibration, uncertainty, and decision thresholds are seldom reported. - Signal scope is narrow (motor-centric); multimodal/non-motor coverage is limited. | - Conduct multi-centre, long-term real-world studies with preregistration, sample-size estimation and attrition handling. - Expand multimodal sensing and non-motor outcomes. - Recommend including external validation, calibration/uncertainty, and decision-curve/threshold metrics as required (or prioritized) reporting elements. |
| **Technical limitation** | |
| - Passive data capture dominates; laboratory/clinic settings over-represented. - Interoperability and standardisation gaps hinder scale-up across devices/sites - On-device constraints for real-time inference (compute/power). | - Promote engineering standardisation and interoperability across devices and pipelines. - Develop lightweight edge models; enable real-time, low-power deployment; strengthen multimodal fusion. - Explore transfer/self-supervised learning to reduce labeled-data dependence |
| **Usability, adherence and equity** | |
| - Wearability/usability burdens; patient adherence and caregiver/clinician workload; digital divide. | - Improve comfort and usability; co-design with users; provide training and literacy support to promote equitable access. - Embed monitoring and feedback into routine workflows to reduce burden. |
| **Economic and policy barriers** | |
| - Unclear reimbursement pathways and economic sustainability. | - Establish reimbursement and regulatory support; accumulate economic and reimbursement evidence to support adoption and sustainability. |
| **Privacy, security and governance** | |
| - Privacy and data-security frameworks are incomplete, hindering multi-centre data sharing. | - Strengthen privacy/data-security governance to enable responsible and ethical data sharing. |
| **Clinical translation and workflow integration** | |
| - Monitoring or assessment dominates; prediction and rehabilitation/feedback are under-represented; closed-loop is rare. - Few externally validated models; limited home/community evidence; weak workflow integration. | - Define actionable intervention thresholds and build evaluate–intervene–feedback–re-evaluate loops. - Prioritize home/community continuous use and multi-centre workflow embedding. |
